# Safety, Tolerability, Pharmacodynamics and Pharmacokinetics of Umeclidinium and Vilanterol Alone and in Combination: A Randomized Crossover Trial

**DOI:** 10.1371/journal.pone.0050716

**Published:** 2012-12-17

**Authors:** Dennis L. Kelleher, Rashmi S. Mehta, Bernadette M. Jean-Francois, Andrew F. Preece, James Blowers, Glenn D. Crater, Paul Thomas

**Affiliations:** 1 Respiratory Medicines Development Center, GlaxoSmithKline, Research Triangle Park, North Carolina, United States of America; 2 GlaxoSmithKline Australia Pty Ltd, Melbourne, Australia; 3 Respiratory Medicines Development Center, GlaxoSmithKline, Stockley Park West, United Kingdom; 4 GlaxoSmithKline, Mississauga, Canada; 5 Medicines Research Unit, GlaxoSmithKline, New South Wales, Australia; Leiden University Medical Center, The Netherlands

## Abstract

Umeclidinium bromide (GSK573719; UMEC), a new long-acting muscarinic receptor antagonist (LAMA), is in development with vilanterol (GW642444; VI), a selective long-acting β_2_ agonist (LABA), as a once-daily inhaled combination therapy for the treatment of chronic obstructive pulmonary disease (COPD). A single dose healthy volunteer study was conducted to assess the safety and tolerability, pharmacodynamics (PD) and pharmacokinetics (PK) of inhaled umeclidinium (500 µg) and vilanterol (50 µg) when administered separately and in combination using a novel dry powder inhaler (NDPI). Co-administration of single inhaled doses of umeclidinium and vilanterol to healthy Japanese subjects was well tolerated and not associated with meaningful changes in systemic exposure or PD effects compared with administration of either compound individually. Pharmacokinetic assessments showed rapid absorption for both drugs (Tmax = 5 min for both umeclidinium and vilanterol) followed by rapid elimination with median tlast of 4–5 h for umeclidinium and median tlast of 1.5–2.0 h for vilanterol. Assessments of pharmacokinetic interaction were inconclusive since for umeclidinium, Cmax following combination was higher than umeclidinium alone but not AUC whereas for vilanterol, AUC following combination was higher than vilanterol alone but not Cmax. There were no obvious trends observed between individual maximum supine heart rate and umeclidinium Cmax or vilanterol Cmax when delivered as umeclidinium 500 µg and vilanterol 50 µg combination or when delivered as umeclidinium or vilanterol alone.

**Trial Registration:**

Clinicaltrials.gov NCT00976144

## Introduction

Umeclidinium (GSK573719; UMEC) is a new inhaled long-acting muscarinic receptor antagonist (LAMA) in development for combination therapy with vilanterol (GW642444; VI), a potent and selective long-acting β_2_ agonist (LABA) [1], as a once daily treatment for chronic obstructive pulmonary disease (COPD). COPD is a major cause of morbidity and mortality worldwide and the direct treatment related costs and lost work and productivity costs pose a major economic burden [2]–[5]. Current treatment guidelines for COPD recommend bronchodilators, usually a β2-adrenoceptor agonist, or a LAMA. If symptoms are not adequately controlled by monotherapy, additional benefit may be provided by combination therapy with different drug classes [6]–[8].

We report the key results of a Phase I trial to evaluate the safety and tolerability, pharmacodynamics (PD) and pharmacokinetics (PK) of umeclidinium and vilanterol as inhaled dose monotherapies and administered concurrently from separate novel dry powder inhaler (NDPI) devices. This study was a randomized, double blind, placebo controlled, four-way crossover study in 16 healthy Japanese male volunteers.

## Methods

### Study Design

This study (protocol number: DB2113208; Clinicaltrials.gov identifier: NCT00976144) was a single center, double-blind, placebo-controlled, four-way randomized crossover trial and was conducted between July and September 2009. The protocol for this trial and supporting CONSORT checklist are available as supporting information; see Checklist S1 and Protocol S1. Patients and investigators were blinded to treatment assignment and the treatments were indistinguishable. Sixteen subjects (not pre-screened for bronchodilator responsiveness) received single inhaled doses of the following treatments: (1) Placebo and placebo; (2) umeclidinium 500 µg and placebo; (3) vilanterol 50 µg and placebo; (4) umeclidinium 500 µg and vilanterol 50 µg via separate NDPI over four treatment periods, each separated by a ≥7 day wash-out. The treatment order was determined by the randomization schedule. The randomization schedule was generated by GlaxoSmithKline Discovery Biometrics using validated internal software (RandALL). Subjects were assigned to one of the four treatment sequences which were based on a Williams Design [9] in accordance with the randomization schedule, prior to the start of the study. Primary endpoint was safety and tolerability, specifically adverse events (AEs), vital signs (heart rate [HR], blood pressure, electrocardiogram (ECG), 24 h Holter monitoring and clinical laboratory assessments. PD endpoints included blood potassium and HR. Secondary endpoints were plasma concentrations of umeclidinium and vilanterol and derived PK parameters. Lung function assessment by spirometry (FEV_1_) was exploratory.

### Subjects

Healthy non-smoking Japanese male volunteers (20–65 years of age) were enrolled. A body weight >45 kg and a body mass index within the range 18 to 28 kg/m2 was required. Subjects were required to have no clinically active and relevant abnormality on a 12-lead ECG or 24 h Holter ECG and to have normal spirometry (forced expiratory capacity in 1 second [FEV_1_]≥80% of predicted, FEV_1_/forced vital capacity [FVC]≥70%) at screening. Subjects with a QTcB >450 milliseconds (msec) or an ECG not suitable for QT measurements were not eligible.

All volunteers provided written informed consent prior to screening. This study was conducted in accordance with WMA Declaration of Helsinki – Ethical Principles for Medical Research Involving Human Subjects [10] at a single center in Australia. The Bellberry Human Research Ethics Committee, 229 Greenhill Rd, Dulwich, South Australia 5065, Australia approved this protocol.

### Sample collection

Blood samples were taken via an indwelling cannula or by direct venipuncture and collected into an ethylene diamine tetraacetic acid tube and immediately placed on water ice. Samples were centrifuged at 1500 rpm for 10 min under chilled conditions. Supernatant plasma was transferred to a 3.6 mL Nunc tube and stored at −80°C before shipment. Samples were batched and shipped frozen on dry ice to a central laboratory for analyses.

### Safety analyses

AE and SAE data were collected and recorded starting on Day 1 and continuing until the end of the confinement period and at follow-up. All safety and tolerability endpoints (AEs, HR, systolic and diastolic blood pressure, 12-Lead ECG [QTc(B) and QTc(F)], lung function FEV_1_, 24 h Holter monitoring including maximum and mean HR and laboratory tests) were summarized. The final statistical analyses were performed after the database had been frozen. Plots of means and 95% CIs for maximum and mean (0–24 h) Holter HRs were produced. Maximum (0–4 h) and weighted mean (0–4 h) of HR (vital signs), QTc(B) and QTc(F) were derived and each of these variables was separately analyzed using a mixed effects model. Subject-level baseline, period-level baseline, period and randomized treatment were fitted as fixed effects and subject was fitted as a random effect.

### Pharmacodynamic analyses

Blood potassium. In addition to inclusion as part of standard laboratory safety assessments, blood potassium, known to be decreased by β2-adrenoceptor agonists, was also monitored to assess the PD effects of vilanterol. Minimum (0–4 h) and weighted mean (0–4 h) for blood potassium were derived and each of these variables was statistically analyzed using a mixed effects model. The model included no baseline (none available). Treatment was fitted as a fixed effect and subject fitted as a random effect.

FEV_1_. Bronchodilation is the desired pharmacodynamic effect for both long-acting muscarinic antagonists and β2-adrenoceptor agonists. Bronchodilation is expressed in asthma and COPD patients as an increase in forced expiratory volume. While bronchodilation in response to a LAMA and/or LABA is not always demonstrable in healthy volunteer subjects, spirometry was included in this study as an exploratory PD assessment. The maximum of three individual readings of FEV_1_ data at serial time points (0–24 h post dose) was analyzed using a mixed effects model. Repeated measures analysis was carried out using subject by period as a blocking effect. The model included subject-level baseline, period-level baseline, period, randomized treatment, time and interaction of treatment and time, subject level baseline and time, period level baseline and time fitted as fixed effects and subject fitted as a random effect.

### Pharmacokinetic analyses

Plasma samples for umeclidinium and vilanterol were analyzed using a validated analytical method based on protein precipitation, followed by high performance liquid chromatography with mass spectrometry (HPLC/MS/MS) analysis. The lower limit of quantification (LLQ) for umeclidinium and vilanterol was 20 pg/mL and 30 pg/mL, respectively using a 100 µL aliquot of human plasma. The higher limit of quantification for umeclidinium and vilanterol was 20,000 pg/mL and 30,000 pg/mL, respectively. For the analytical method, quality control samples (QC), containing umeclidinium and vilanterol at 3 different concentrations and stored with study samples, were analyzed with each batch of samples against separately prepared calibration standards. For the analysis to be acceptable, no more than one-third of the QC results were to deviate from the nominal concentration by more than 15%, and at least 50% of the results from each QC concentration were to be within 15% of nominal. The applicable analytical runs met all predefined run acceptance.

Concentrations of umeclidinium and vilanterol in plasma were summarized by treatment and planned time. The derived PK parameters AUC(0–0.25), AUC(0–2), AUC(0–t), AUC(0–∞), AUClast, Cmax, t½, tmax and tlast were summarized for the analyte umeclidinium and the parameters AUC(0–0.25), AUC(0–0.5), AUC(0–2), Cmax, t½, tmax and tlast were summarized for the analyte vilanterol. Vilanterol AUC(0–∞) was planned, however due to limitations in the plasma concentration profile only AUC(0–2) and previous AUCs were possible.

For the analyte umeclidinium, log transformed values of AUC(0–∞) and Cmax were analyzed and for the analyte vilanterol, log transformed values of AUC(0–0.25), AUC(0–2) and Cmax were analyzed using a mixed effects model. Treatment was fitted as a fixed effect and subject was fitted as a random effect.

### Sample size considerations

Sixteen subjects were planned to be recruited into the study and a minimum of 12 subjects were needed to complete the study. With 12 subjects, assuming no difference between the treatment groups, it was estimated (based on prior studies [11]) that the lower and upper bounds of the 95% confidence interval (CI) for the difference between test and reference treatments of maximum heart rate (0–4 h) would be within approximately 5.857 bpm of the point estimate.

## Results

### Baseline characteristics and subject disposition

**Figure 1:**
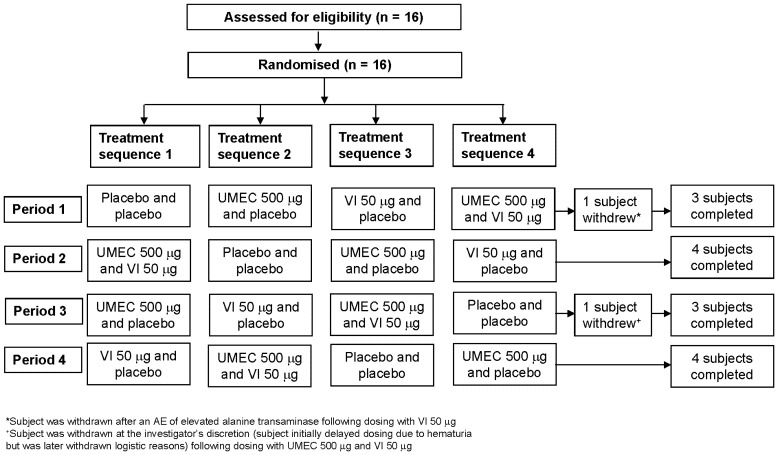
CONSORT diagram.

Sixteen healthy male subjects of Japanese heritage were enrolled (Figure 1). The subject population had a mean (range) age of 29 (21–58) years and mean (range) body mass index of 21.7 (18.5 –25.1) kg/m^2^. Fourteen subjects completed the study. One subject was withdrawn from the study at the investigator's discretion (the subject initially delayed dosing due to hematuria but was later withdrawn in Period 3 for logistic reasons) after dosing with umeclidinium 500 µg and vilanterol 50 µg. One subject was withdrawn due to an AE of elevated alanine transaminase (ALT) first observed on the Day −1 (pre-dose) visit during Period 2.

### Safety and tolerability

Study treatments were safe and well tolerated and no serious adverse events (SAEs) or deaths were reported. Thirteen AEs were reported in eight (50%) of the subjects over the four treatment periods (Table 1). All AEs were of mild intensity and none were considered by the investigator to be possibly related to study drug. Musculoskeletal pain and muscle strain (2 each) were the only AEs reported by more than one subject. There were no clinically significant findings observed in any subject for vital signs, ECGs, Holter or hematology parameters. One subject dosed with vilanterol 50 µg in Period 1 had high ALT, aspartate transaminase (AST) and creatine kinase values on the Day −1 (pre-dose) visit during Period 2. Elevated ALT was recorded as an AE. AST and creatine kinase levels were recorded as Chemistry Laboratory Data for Subjects with Abnormalities of Potential Clinical Importance but were marked as not being of Clinical Importance. On inquiry the subject admitted to recent strenuous exercise resulting in strained muscles but he was otherwise asymptomatic. Repeat investigation of liver function tests the following day indicated continued elevation. The pre-specified liver chemistry stopping criterion was met (ALT>3×ULN) and investigational product was stopped. Subsequently the subject was withdrawn from the study by the investigator. The event was of mild intensity, not considered study drug related by the investigator and resolved following treatment discontinuation.

**Table 1:** Summary of number of subjects reporting all AEs on treatment.

Adverse Event	Placebo (n = 14)	UMEC 500 µg (n = 15)	VI 50 µg (n = 16)	UMEC 500 µg/VI 50 µg (n = 15)	Total (n = 16)
Any event n (%)	1 (7%)	6 (40%)	1 (6%)	2 (13%)	7 (44%)
Musculoskeletal pain	0	2 (13%)	0	0	2 (13%)
Muscle strain	0	1 (7%)	1 (6%)	0	2 (13%)
Joint sprain	0	1 (7%)	0	0	1 (6%)
Dizziness	0	1 (7%)	0	0	1 (6%)
Headache	0	0	0	1 (7%)	1 (6%)
Presyncope	1 (7%)	0	0	0	1 (6%)
Chest discomfort	0	1 (7%)	0	0	1 (6%)
Gastroenteritis	0	1 (7%)	0	0	1 (6%)
ALT	0	0	1 (6%)	0	1 (6%)
Cough	0	0	0	1 (7%)	1 (6%)

Note: Total is the total number of subjects experiencing the event not a total number of events.

UMEC = umeclidinium; VI = vilanterol.

There were no hematology or serum clinical chemistry treatment-related trends identified over time (samples taken at Day −1 and 24 h post-dose) in any of the treatment periods.

The average maximum heart rates (HR) and weighted mean HR are shown in Table 2. There were modest increases in HR. For the average maximum HR (0–4 h), increases were observed for umeclidinium, vilanterol, and the umeclidinium/vilanterol combination treatment compared with placebo. When the combination was compared with each monotherapy, average increases for umeclidinium/vilanterol compared with umeclidinium and for umeclidinium/vilanterol compared with vilanterol were observed. For weighted mean HR (0–4 h) similar results were obtained but with smaller increases. The greatest increase was for the vilanterol treatment compared with placebo. Small increases were observed in the other treatment comparisons except for umeclidinium/vilanterol compared with vilanterol alone where a slight decrease was observed.

**Table 2:** Summary of heart rate parameter adjusted means.

Treatment comparison	Maximum heart rate (0–4 h)			Weighted mean heart rate (0–4 h)		
	Adjusted means (bpm)		Difference (95% CI) (bpm)	Adjusted means (bpm)		Difference (95% CI) (bpm)
	Test	Reference		Test	Reference	
Umeclidinium - Placebo	62.2	60.1	2.1 (−2.1, 6.2)	56.7	56.2	0.5 (−2.1, 3.1)
Vilanterol – Placebo	64.7	60.1	4.6 (0.5, 8.7)	59.0	56.2	2.8 (0.2, 5.4)
Umeclidinium/Vilanterol - Placebo	65.0	60.1	4.8 (0.6, 9.1)	58.4	56.2	2.2 (−0.5, 4.9)
Umeclidinium/Vilanterol - Umeclidinium	65.0	62.2	2.8 (−1.2, 6.8)	58.4	56.7	1.7 (−0.8, 4.3)
Umeclidinium/Vilanterol - Vilanterol	65.0	64.7	0.3 (−3.7, 4.2)	58.4	59.0	−0.6 (−3.1, 1.9)

QT interval results were evaluated utilizing standard heart rate-corrected interval (QTc), Bazett's (B) and Fridericia's (F) formulas (Table 3). Most QT interval derived parameters showed modest increases. With regards to average maximum QTc(B) (0–4 h) observations, increases were observed for umeclidinium, vilanterol, and the umeclidinium/vilanterol combination treatment compared with placebo. When the combination was compared with each monotherapy, average increases for umeclidinium/vilanterol compared with umeclidinium and for umeclidinium/vilanterol compared with vilanterol were observed.

**Table 3:** Summary of QTc adjusted means.

Treatment comparison	QTc(B) (0–4 h)		QTc(F) (0–4 h)	
	Maximum difference (95% CI) (msec)	Weighted mean difference (95% CI) (msec)	Maximum difference (95% CI) (msec)	Weighted mean difference (95% CI) (msec)
Umeclidinium - Placebo	4.2 (−4.3, 12.7)	−1.1 (−5.7, 3.6)	1.2 (−5.5, 7.9)	−1.5 (−5.1, 2.1)
Vilanterol – Placebo	15.4 (7.0), 23.8)	8.5 (3.9, 13.1)	8.3 (1.6, 15.0)	5.1 (1.5, 8.6)
Umeclidinium/Vilanterol - Placebo	20.3 (11.8, 28.8)	7.2 (2.5, 11.8)	11.3 (4.6, 18.1)	3.9 (0.4, 7.5)
Umeclidinium/Vilanterol - Umeclidinium	16.1 (7.8, 24.3)	8.2 (3.7, 12.8)	10.1 (3.6, 16.7)	5.4 (2.0, 8.9)
Umeclidinium/Vilanterol - Vilanterol	4.9 (−3.4, 13.2)	−1.3 (−5.9, 3.2)	3.1 (−3.4, 9.5)	−1.1 (−4.6, 2.3)

Average maximum QTc(F) (0–4 h) observations included increases for umeclidinium, vilanterol and the umeclidinium/vilanterol combination treatment compared with placebo. When the combination was compared with each monotherapy, average increases for umeclidinium/vilanterol compared with umeclidinium and for umeclidinium/vilanterol compared with vilanterol were observed.

For weighted mean QTc(B) (0–4 h) observations increases were observed for the comparisons of vilanterol and the umeclidinium/vilanterol combination treatment compared with placebo. For the comparison of umeclidinium/vilanterol with umeclidinium, an increase was observed. The other two comparisons were marginally decreased. All observations for weighted mean QTc(F) (0–4 h) comparisons were marginal.

### Pharmacodynamics

**Figure 2:**
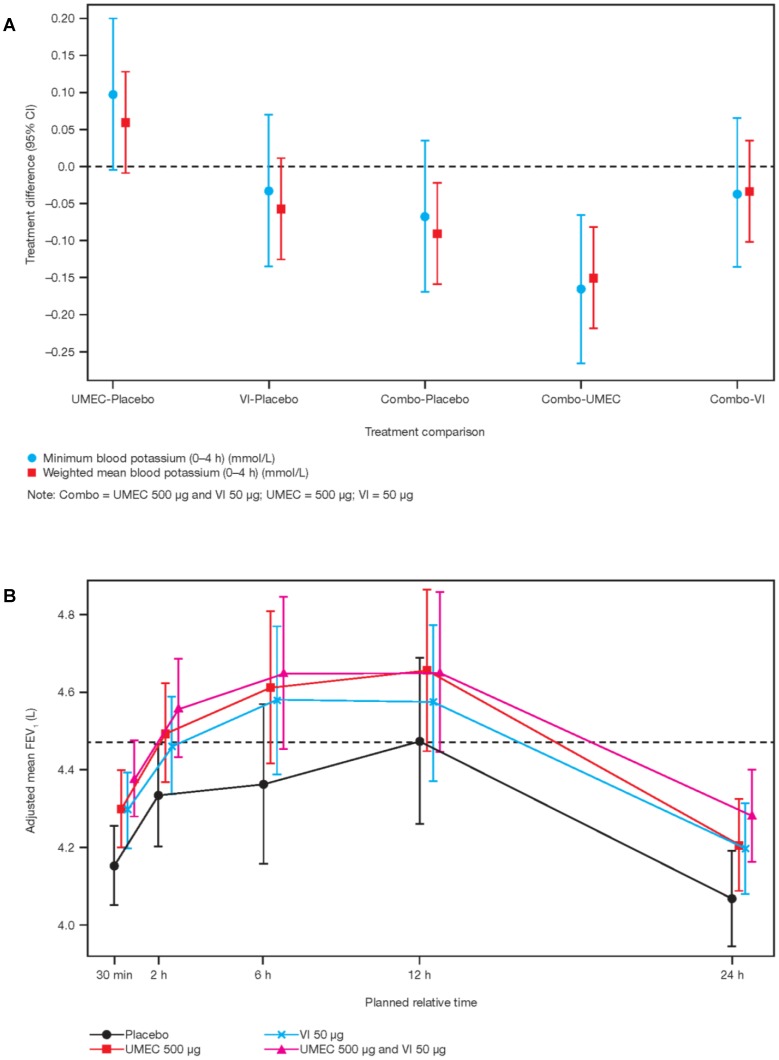
Pharmacodynamic analyses. (a) Analysis of derived blood potassium parameters. (b) Plot of adjusted means FEV_1_ time profile.

A plot of the differences for minimum blood potassium (0–4 h) and weighted mean potassium (0–4 h) is provided in Figure 2a. Treatment with vilanterol monotherapy compared with placebo or umeclidinium/vilanterol in combination relative to placebo or to either monotherapy resulted in small average decreases in minimum blood potassium (0–4 h). However, the 95% CI crossed 0.0 except for umeclidinium/vilanterol compared with umeclidinium monotherapy which was −0.17 mmol/L (95% CI:−0.27, −0.07). Umeclidinium monotherapy relative to placebo resulted in a small increase in minimum potassium (0–4 h) relative to placebo of 0.10 mmol/L (95% CI: 0.00, 0.20). Weighted mean potassium (0–4 h), followed a similar pattern to minimum blood potassium (0–4 h) with an increase being observed only in the comparison of umeclidinium with placebo. The other treatment comparisons showed decreases, with the greatest decrease [−0.15 mmol/L (95% CI: −0.22,−0.08)] being in the comparison between the umeclidinium/vilanterol combination vs. umeclidinium monotherapy.

Mean predose FEV_1_ values (L) were similar across treatments: placebo, 3.996 (95% CI: 3.706, 4.287); umeclidinium monotherapy, 4.078 (95% CI: 3.763, 4.393); vilanterol monotherapy, 4.134 (95% CI: 3.853, 4,414); umeclidinium/vilanterol combination, 4.045 (95% CI: 3.766, 4.324). Adjusted means from serial time point analysis showed that FEV_1_ values were higher for all treatment periods compared with placebo (Figure 2b). The umeclidinium/vilanterol combination treatment at 6 h showed the largest difference relative to placebo with an FEV_1_ difference in adjusted mean of 287 mL (95% CI: 14 mL, 560 mL).

### Pharmacokinetics

#### Umeclidinium

The bioanalytical method used to quantify plasma umeclidinium concentrations had a LLQ of 20 pg/mL; such that full characterization of the PK profile of umeclidinium following the single dose administration was not possible. Overall 42% of samples (165/390) were non-quantifiable (NQ). Approximately 46% (89 out of 195) umeclidinium plasma concentrations were NQ following umeclidinium monotherapy and 39% (76 out of 195) of plasma concentrations were NQ following umeclidinium 500 µg/vilanterol 50 µg in combination. Moderate to large between-subject variability was observed for both treatments with values for between-subject coefficient of variation (CVb) ranging from 50% to 421% for maximum observed concentration (Cmax) and 47% to 162% for the area under the concentration-time curve (AUC).

Umeclidinium plasma concentrations and derived PK parameter results are summarized in Table 4 along with the ratio of adjusted geometric means. Umeclidinium was rapidly absorbed with all of the plasma Cmax occurring at 5 min followed by a rapid decline. The ratio of the adjusted geometric means of AUC(0–∞) was not different for systemic exposure when delivered as umeclidinium/vilanterol in combination compared with umeclidinium administered as monotherapy. The ratio for Cmax was higher when delivered in the combination treatment compared with umeclidinium monotherapy.

**Table 4:** Pharmacokinetics of single dose umeclidinium.

Parameter	Adjusted geometric mean (90% CI)^a^		Ratio of adjusted geometric means (90% CI)^b^
	UMEC 500 µg (n = 15)	UMEC/VI 500/50 µg (n = 15)	UMEC/VI 500/50 µg vs. UMEC 500 µg
AUC(0–0.25) (h•pg/mL)	149.7* (118.2, 189.7))	180.9 (145.4, 225.2)	1.21 (0.93, 1.56)
AUC(0–2) (h•pg/mL)	391.1* (306.3, 499.4)	416.4 (332.8, 520.9)	1.06 (0.80, 1.41)
AUC(0–4) (h•pg/mL)	475.8* (364.7, 620.8)	518.1 (404.2, 664.1)	1.09 (0.82, 1.44)
AUC(0-∞) (h•pg/mL)	575.7** (419.6, 789.8)	623.7 (474.1, 820.6)	1.08 (0.74, 1.59)
Cmax (pg/mL)	995.9* (776.0, 1278.1)	1299.0 (1026.0, 1644.7)	1.30 (1.04, 1.64)
t½ (h)^c^	1.56† (1.29, 1.90)	1.78 (1.17, 2.70)	NC
tmax (h)^d^	0.08‡ (0.08, 0.13)	0.08 (0.08, 0.08)	NC
tlast (h)^d^	4.85‡ (1.00, 8.00)	5.47 (1.00, 16.00)	NC

AUC(0–t) = area under concentration-time curve from time 0 to time of last quantifiable concentration; Cmax = maximum observed plasma concentration; NC = not calculated; tlast = last timepoint where the concentration is above the limit of quantification; tmax = time of maximum observed plasma concentration; UMEC = umeclidinium; VI = Vilanterol;

a Summary statistics derived following Cmax imputation with ½ lower limit of quantification (LLQ) (10 pg/mL) and AUC imputation with ½ lowest observed value for the parameter across treatments;

* Data from two subjects are imputed;

** Data from three subjects are imputed,

† n = 12,

‡ n = 13.

b UMEC/VI vs UMEC ratio analysis done without imputing the non-quantifiable (NQ) results.

c Presented as geometric mean and 95% CI.

d Presented as median and range.

#### Vilanterol

The bioanalytical method used to quantify plasma vilanterol concentrations had a LLQ was 30 pg/mL; such that full characterization of the PK profile of vilanterol following single dose administration of vilanterol monotherapy or concurrently with umeclidinium was not possible. Overall 60% of samples (240/403) were NQ following the single dose administration. Approximately 61% (127 of 208) vilanterol plasma concentrations were NQ following vilanterol monotherapy and about 58% (113 of 195) vilanterol plasma concentrations were NQ following vilanterol 50 µg in combination with 500 µg of umeclidinium. Moderate to high between-subject variability was observed for both treatments with values for the CVb% ranging from 44% to 86% for Cmax and 37% to 56% for AUC.

Vilanterol plasma concentrations and derived PK parameter results are summarized by treatment in Table 5 with the ratio of adjusted geometric means. Vilanterol was rapidly absorbed with most of the plasma Cmax occurring at 5 min followed by a rapid decline. The analysis showed there was no difference in vilanterol Cmax when delivered as umeclidinium/vilanterol in combination compared with vilanterol monotherapy. The ratio for AUC showed higher exposure when umeclidinium and vilanterol were administered concurrently compared with vilanterol administered alone.

**Table 5:** Pharmacokinetics of single dose vilanterol.

Parameter	Adjusted Geometric Mean (90% CI)^a^		Ratio of Adjusted Geometric Means (90% CI)^b^
	VI 50 µg (N = 16)	UMEC/VI 500/50 µg (N = 15)	UMEC/VI 500/50 µg vs. VI 50 µg
AUC(0–1) (h•pg/mL)	208.0 (181.8, 237.9)	252.3* (218.2, 291.8)	1.21 (1.02, 1.44)
AUC(0-∞) (h•pg/mL)	254.1* (215.5, 299.7)	352.5* (296.2, 419.4)	1.39 (1.07, 1.80)
Cmax (pg/mL)	495.9 (386.2, 636.8)	499.9 (385.8, 647.8)	1.01 (0.70, 1.45)
t½ (h)^c^	0.42* (0.36, 0.49)	0.71* (0.52, 0.97)	NC
tmax (h)^d^	0.08 (0.08, 0.10)	0.08 (0.08, 0.08)	NC
tlast (h)^d^	1. 62 (1.00, 4.00)	1.91 (0.08, 5.00)	NC

UMEC = umeclidinium; VI = Vilanterol; AUC (0-t) = area under concentration-time curve from time 0 to time of last quantifiable concentration; Cmax = maximum observed plasma concentration; tmax = time of maximum observed plasma concentration; tlast = last time point where the concentration is above the limit of quantification, CI = confidence interval; NC = not calculated.

a Summary statistics derived following Cmax imputation with ½ LLQ (15 pg/mL) and AUC imputation with ½ lowest observed value for the parameter across treatments;

* Data from one subject is imputed.

b UMEC/VI vs VI ratio analysis done without imputing the NQ results.

c Presented as geometric mean and 95% CI.

d Presented as median and range.

### Relationship between PK and PD parameters

**Figure 3:**
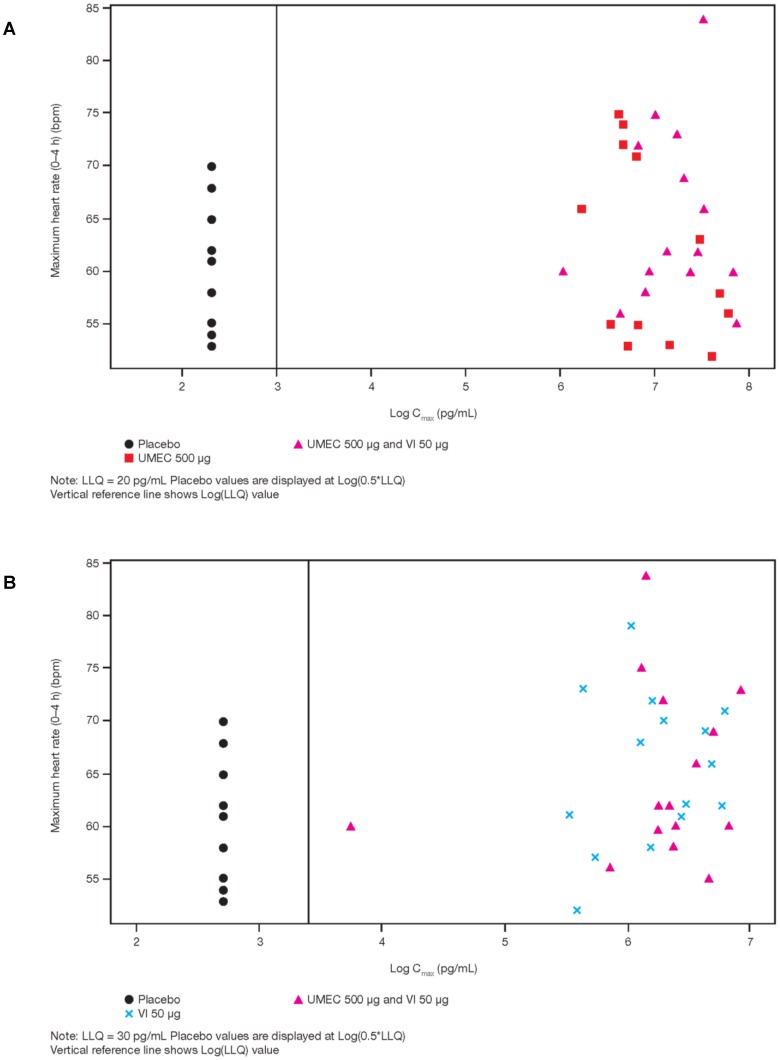
Plot of individual maximum (0–4 h) HR versus Cmax. (a) Umeclidinium log Cmax. (b) Vilanterol log Cmax.

Pooled scatter plots of the PD variable ‘individual maximum for supine HR’ versus umeclidinium Cmax and vilanterol Cmax are shown in Figure 3. No obvious trends between individual maximum HR and umeclidinium Cmax (panel a) or vilanterol Cmax (panel b) when administered as umeclidinium/vilanterol combination or as umeclidinium or vilanterol administered as monotherapy were noted on

visual inspection.

## Discussion

We report the results of the first time in human study providing clinical data for the LAMA/LABA combination of umeclidinium/vilanterol. This single dose study assessed the safety and tolerability, PD and PK of umeclidinium and vilanterol as monotherapies and in combination. The study was conducted in male subjects of Japanese heritage.

Single inhaled doses of umeclidinium 500 µg, vilanterol 50 µg and the combination were safe and well tolerated. There were no deaths or SAEs reported. A total of 13 AEs were reported by 8 subjects (50%) and all AEs were of mild intensity. There were no clinically significant or relevant changes in vital signs, hematology, and clinical chemistries that were attributed to the study drug. There were also no clinically significant 12-lead ECG or 24 h Holter ECG abnormalities observed during the study following dosing of study medication. No significant episodes of arrhythmia were observed. There were some minor elevations in HR and ECG parameters that were not considered of potential clinical importance.

β agonists are known to contribute to a lowering of blood potassium levels [12], [13]. PD analysis of blood potassium parameters showed small decreases in minimum and weighted mean blood potassium levels over the 4 h period for all treatment comparisons except for umeclidinium compared with placebo where there was an increase of 0.10 mmol/L. This increase may have influenced the larger decrease of the umeclidinium/vilanterol compared with umeclidinium treatment which was the only treatment comparison decrease for which the 95% CI did not cross 0.0.

Increases in FEV_1_ following bronchodilator inhalation has been demonstrated in healthy non smoking adult subjects [14], [15]. The FEV_1_ exploratory analysis in our study revealed a slightly higher adjusted mean FEV_1_ for all active treatments compared to placebo, with the highest increase in FEV_1_ being the umeclidinium and vilanterol concurrent administration at 6 h.

The concurrent administration of umeclidinium and vilanterol resulted in a 30% higher umeclidinium Cmax than umeclidinium alone although the treatment ratio for AUC parameters, with the exception of AUC_(0–0.25)_, showed no difference. It is important to note that no trends were observed between individual change from baseline maximum for supine HR and umeclidinium Cmax when delivered as umeclidinium vilanterol administered concurrently or when delivered as umeclidinium alone.

There was no difference in vilanterol Cmax when delivered as umeclidinium and vilanterol administered concurrently compared with vilanterol alone and no trends were observed between individual change from baseline maximum for supine HR and vilanterol Cmax when delivered as umeclidinium and vilanterol administered concurrently or vilanterol alone. AUC parameters indicated that the concurrent administration of umeclidinium and vilanterol resulted in an up to 39% higher systemic exposure to vilanterol when compared to vilanterol alone.

The factors contributing to the approximately 30% higher umeclidinium Cmax and 39% higher vilanterol AUC when administered concurrently in this single dose study have not been clearly identified. However, a potentially important aspect of the study conduct was that within a treatment period the order of dosing using the 2 separate inhalers was not pre-defined. There was also a formulation difference in treatments due to increased magnesium stearate exposure using two separate inhalers for the concurrent administration compared with umeclidinium and vilanterol alone. Regardless of the factors contributing to the increased exposure, the results of a repeat dose combination study (GlaxoSmithKline protocol: DB2113950; Clinicaltrials.gov identifier: NCT01128634) indicated that when administered simultaneously in a single inhaler device, vilanterol did not have an effect on systemic exposure of umeclidinium following single or repeat doses (R. Mehta et al., unpublished data). Thus concerns of a potential PK interaction have not been substantiated and the observations of increased exposure of umeclidinium Cmax and vilanterol AUC from the combination reported in this study are not considered clinically meaningful.

With regard to study limitations, the sample size of the study population was small. Although the study aimed to recruit 16 subjects and have a minimum of 12 subjects complete the study, a larger population would have allowed for additional analysis and statistical determinations.

In conclusion, administration of single inhaled doses of umeclidinium (500 µg) alone, vilanterol (50 µg) alone and administered concurrently to healthy Japanese male subjects was safe and well tolerated and not associated with meaningful changes in systemic exposure or PD effects compared with administration of either compound alone.

## Supporting Information

Checklist S1 **CONSORT Checklist.**
(DOC)

Protocol S1 **Trial Protocol.**
(PDF)
