# Supplementary material for: Safety, Tolerability, Pharmacodynamics and Pharmacokinetics of Umeclidinium and Vilanterol Alone and in Combination: A Randomized Crossover Trial
Source: PLoS One. 2012 Dec 17;7(12):e50716. doi: 10.1371/journal.pone.0050716 (PMC3524248; doi:10.1371/journal.pone.0050716)

Division: Worldwide Development
Retention Category: GRS019
Information Type: Clinical Pharmacology Protocol

**Title:**	A single centre, randomised, placebo-controlled, four-way cross over study to assess the safety, tolerability, pharmacodynamics and pharmacokinetics of single inhaled doses of GSK573719 and GW642444 as monotherapies and concurrently in healthy Japanese subjects.

Compound Number: GSK573719+GW642444

Effective Date: 04-JUN-2009

Description: GW642444 is a potent and selective long-acting β_2 agonist; GSK573719 is a long-acting, inhaled, muscarinic receptor antagonist (or anticholinergic) bronchodilator. Both are in development as once daily (QD) monotherapies for the treatment of Chronic Obstructive Pulmonary Disease (COPD). Development of these two inhaled drugs as a combination therapy is also planned and would have potential for improved efficacy and patient benefit as they both work through different receptor pathways and the combined bronchodilatory effect might be additive.

This study is a randomised, double blind, placebo-controlled, four-way crossover study which will assess the safety, tolerability, pharmacodynamics (PD) and pharmacokinetics (PK) of GSK573719 and GW642444 in sixteen healthy Japanese subjects. Subjects will receive four possible treatments as single inhaled doses, receiving the two monotherapies separately, the monotherapies concurrently, and placebo.

Blood samples for PK analysis will be taken at regular intervals after dosing. Safety will be assessed by measurement of heart rate, blood pressure, ECG and twenty-four hour Holter monitoring, potassium, safety laboratory data and review of adverse events.

Subject: Muscarinic Receptor Antagonist, Anticholinergic, β_2 agonist, LAMA, LABA, healthy Japanese subjects, safety, tolerability, pharmacokinetics, pharmacodynamics, GSK573719, GW642444.

Author(s):

Jean-Francois, Bernadette MJ [redacted] Preece, Andrew F [redacted], Kelleher Dennis L [redacted] Crater, Glenn D [redacted] , Donald Alison, [redacted], Mehta, Rashmi [redacted]

Copyright 2009 the GlaxoSmithKline group of companies. All rights reserved. Unauthorised copying or use of this information is prohibited.

CONFIDENTIAL GM2009/00078/00
DB2113208

[redacted]

Glenn D. Crater M.D. FCCP — **Date**
PPL, Global Clinical Respiratory Medicine
Development Centre, GlaxoSmithKline

SPONSOR/MEDICAL MONITOR INFORMATION PAGE

Medical Monitor and Sponsor Contact Information:

Role	Name D	ay Time Phone Number	After-hours Phone/Cell/ Pager Number	Fax Number	GSK Address
Primary Medical Monitor	Glenn Crater, MD	[redacted]	[redacted]	[redacted]	[redacted]
Secondary Medical Monitor	[redacted]	[redacted]	[redacted]	[redacted]	[redacted]

Sponsor Registered Address:

GlaxoSmithKline Research & Development Limited
980 Great West Road
Brentford
Middlesex, TW8 9GS
UK

In some countries, the clinical trial sponsor may be the local GlaxoSmithKline affiliate company (or designee). If applicable, the details of the alternative Sponsor and contact person in the territory will be provided to the relevant regulatory authority as part of the clinical trial application.

Regulatory Agency Identifying Number(s): Not applicable

INVESTIGATOR PROTOCOL AGREEMENT PAGE

I confirm agreement to conduct the study in compliance with the protocol.

- I acknowledge that I am responsible for overall study conduct. I agree to personally conduct or supervise the described clinical study.
- I agree to ensure that all associates, colleagues and employees assisting in the conduct of the study are informed about their obligations. Mechanisms are in place to ensure that site staff receive the appropriate information throughout the study.

Investigator Name:	
Investigator Address:	
Investigator Phone Number:	
Investigator Signature	Date

TABLE OF CONTENTS

Page
ABBREVIATIONS . . . 8
1. INTRODUCTION . . . 12
1.1. Background . . . 12
1.1.1. COPD and clinical management of the disease . . . 12
1.1.2. GSK573719 . . . 12
1.1.3. GW642444 . . . 13
1.1.4. GSK573719 and GW642444 concurrent dosing . . . 14
1.1.5. Magnesium Stearate . . . 14
1.2. Rationale . . . 15
1.2.1. Study Rationale . . . 15
1.2.2. Dose Rationale . . . 15
1.3. Summary of Risk Management . . . 17
2. OBJECTIVE(S) . . . 17
2.1. Primary . . . 17
2.2. Secondary . . . 17
2.3. Exploratory . . . 17
3. ENDPOINT(S) . . . 18
3.1. Primary . . . 18
3.2. Secondary . . . 18
3.3. Exploratory . . . 18
4. INVESTIGATIONAL PLAN . . . 18
4.1. Study Design Description . . . 18
4.2. Discussion of Design . . . 19
4.3. Treatment Assignment . . . 19
4.4. Investigational Product Dosage/Administration . . . 20
4.5. Stopping Criteria . . . 21
4.5.1. Stopping Safety Criteria . . . 21
4.6. Time and Events Table . . . 23
5. STUDY POPULATION . . . 25
5.1. Number of Subjects . . . 25
5.2. Eligibility Criteria . . . 25
5.2.1. Inclusion Criteria . . . 25
5.2.2. Exclusion Criteria . . . 26

5.2.3. Other Eligibility Criteria Considerations 28
6. DATA ANALYSIS AND STATISTICAL CONSIDERATIONS 28
6.1. Hypotheses and Treatment Comparisons 28
6.2. Sample Size Considerations 28
6.2.1. Sample Size Assumptions 28
6.2.2. Sample Size Sensitivity 29
6.2.3. Sample Size Re-estimation 30
6.3. Data Analysis Considerations 30
6.3.1. Interim Analysis 30
6.3.2. Final Analyses 30
7. STUDY ASSESSMENTS AND PROCEDURES 32
7.1. Demographic/Medical History Assessments 32
7.2. Safety 33
7.3. Alcohol, Cotinine and Drugs of Abuse Tests 36
7.4. Pregnancy 36
7.4.1. Action to be taken if pregnancy occurs in a female partner of a male study subject 36
7.5. Pharmacokinetics 37
7.5.1. Blood Sample Collection 37
7.5.2. Sample Analysis 37
8. LIFESTYLE AND/OR DIETARY RESTRICTIONS 37
8.1. Contraception Requirements 37
8.2. Meals and Dietary Restrictions 37
8.3. Caffeine, Alcohol, and Tobacco 38
8.4. Activity 38
9. CONCOMITANT MEDICATIONS AND NON-DRUG THERAPIES 38
9.1. Permitted Medications 38
9.2. Prohibited Medications 39
9.3. Non-Drug Therapies 39
10. COMPLETION OR EARLY WITHDRAWAL OF SUBJECTS 39
10.1. Subject Completion 39
10.2. Subject Withdrawal Criteria 39
10.3. Subject Withdrawal Procedures 39
10.3.1. Subject Withdrawal from Study 39
10.4. Treatment After the End of the Study 39
10.5. Screen and Baseline Failures 40

11. INVESTIGATIONAL PRODUCT(S) 40
11.1. Blinding 40
11.2. Packaging and Labeling 40
11.3. Preparation/Handling/Storage/Accountability 40
11.4. Assessment of Compliance 41
11.5. Treatment of Investigational Product Overdose 42

12. ADVERSE EVENTS (AE) AND SERIOUS ADVERSE EVENTS (SAE) . . 43
12.1. Definition of Adverse Events 43
12.2. Definition of Serious Adverse Events 44
12.3. Method of Detecting AEs and SAEs 45
12.4. Recording of AEs and SAEs 46
12.5. Evaluating AEs and SAEs 46
12.5.1. Assessment of Intensity 46
12.5.2. Assessment of Causality 46
12.6. Follow-up of AEs and SAEs 47
12.7. Prompt Reporting of SAEs to GSK 47
12.8. Regulatory Reporting Requirements For SAEs 48

13. LIVER CHEMISTRY FOLLOW-UP PROCEDURES 48

14. STUDY CONDUCT CONSIDERATIONS 50
14.1. Regulatory and Ethical Considerations, Including the Informed Consent Process 50
14.2. Quality Control (Study Monitoring) 50
14.3. Quality Assurance 51
14.4. Study and Site Closure 51
14.5. Records Retention 52
14.6. Provision of Study Results and Information to Investigators 52
14.7. Data Management 53

15. REFERENCES 54

APPENDICES 55
APPENDIX 1: LIVER SAFETY ALGORITHMS 55

ABBREVIATIONS

Ae	Urinary recovery of unchanged drug
Ae(0-x)	Urinary recovery of unchanged drug up to fixed nominal time-point x
Ae(0-∞)	Complete urinary recovery of unchanged drug up to time of last measurable urinary concentration
Ae(0-τ)	Urinary recovery over a dosing interval
AE	Adverse Event
ALT	Alanine aminotransferase (SGPT)
ANOVA	Analysis of Variance
AST	Aspartate aminotransferase (SGOT)
AUC	Area under concentration-time curve
AUC(0-∞)	Area under the concentration-time curve from time zero (pre-dose) extrapolated to infinite time
%AUCex	Percentage of AUC(0-∞) obtained by extrapolation
AUC(0-x)	Area under the concentration-time curve from zero (pre-dose) to some fixed nominal time x
AUC(0-t)	Area under the concentration-time curve from time zero (pre-dose) to last time of quantifiable concentration within a subject across all treatments
AUC(0-τ)	Area under the concentration-time curve over the dosing interval
β-HCG	Beta-Human Chorionic Gonadotropin
BA	Bioavailability
BE	Bioequivalence
BMI	Body mass index
BP	Blood pressure
BPM	Beat Per Minute
BQL	Below the quantification limit
BUN	Blood urea nitrogen
CBC	Complete blood count
CI	Confidence Interval
CIB	Clinical Investigator’s Brochure
CLr	Renal clearance
CL	Systemic clearance of parent drug
CL/F	Apparent clearance following oral dosing
Cmax	Maximum observed concentration
Cmin	Minimum observed concentration
Cτ	Pre-dose (trough) concentration at the end of the dosing interval
Ct	Last observed quantifiable concentration
CDMP	Clinical Document Management and Publishing
CO_2	Carbon dioxide
COPD	Chronic obstructive pulmonary disease
CPDM	Clinical Pharmacology and Discovery Medicine
CPDS	Clinical Pharmacology Data Sciences
CPK	Creatine phosphokinase
CPKMS	Clinical Pharmacokinetics Modelling & Simulation

CPSR	Clinical Pharmacology Study Report
CP-RAP	Clinical Pharmacology Reporting and Analysis Plan
CRF	Case Report Form
CRO	Contract Research Organization
CRU	Clinical Research Unit
CSSO	Clinical Science and Study Operations
CV	Coefficient of variance
DB	Discovery Biometrics
DBP	Diastolic blood pressure
DDS	Drug Development Sciences
DILI	Drug Induced Liver Injury
DMPK	Drug Metabolism and Pharmacokinetics
DNA	Deoxyribonucleic acid
ECG	Electrocardiogram
EDC	Electronic data capture
EISR	Expedited Investigator Safety Report
Fabs	Absolute bioavailability of drug determined following extravascular and intravascular dosing
FDA	Food and Drug Administration
FEV_1	Forced expiratory volume in 1 second
Frel	Relative bioavailability of drug determined between two formulations of the same drug following similar or different extravascular route of administration
FSH	Follicle Stimulating Hormone
FTIH	First time in humans
GCP	Good Clinical Practice
GCSP	Global Clinical Safety and Pharmacovigilence
GGT	Gamma glutamyltransferase
GLP	Good Laboratory Practice
GLS	Geometric Least-Squares
GSK	GlaxoSmithKline
HBsAg	Hepatitis B surface antigen
hCG	Human chorionic gonadotropin
HIV	Human Immunodeficiency Virus
h/hr	Hour(s)
HR	Heart rate
HWE	Hardy-Weinberg Equilibrium
IB	Investigator’s Brochure
ICH	International Conference on Harmonization of Technical Requirements for Registration of Pharmaceuticals for Human Use
IDMC	Independent Data Monitoring Committee
IDSL	Integrated Data Standards Library
IEC	Independent Ethics Committee
IND	Investigational New Drug
IP	Investigational Product
IRB	Institutional Review Board

IU	International Unit
IV	Intravenous
Kg	Kilogram
λz	Terminal phase rate constant
L	Liter
LABA	Long-acting β_2 agonist
LAMA	Long-acting muscarinic antagonist
LFTs	Liver function tests
ln	Naperian (natural) logarithm
LOQ	Limit of quantification
LLQ	Lower limit of quantification
µg	Microgram
µL	Microliter
mAChR	Muscarinic acetylcholine receptor
MAT	Mean absorption time
MCH	Mean corpuscular hemoglobin
MCHC	Mean corpuscular hemoglobin concentration
MCV	Mean corpuscular volume
MedDRA	Medical Dictionary for Regulatory Activities
Mg	Milligrams
mL	Milliliter
MRT	Mean residence time
MSDS	Material Safety Data Sheet
msec	Milliseconds
NQ	Non-quantifiable concentration measured as below LLQ
PD	Pharmacodynamic
PGx	Pharmacogenetics
PK	Pharmacokinetic
PSRI	Periodic Safety Reports for Investigators
QC	Quality control
QD	Once daily
QTcB	QT duration corrected for heart rate by Bazett's formula
QTcF	QT duration corrected for heart rate by Fridericia's formula
RAP	Reporting and Analysis Plan
RBA	Relative Bioavailability
RBC	Red blood cells
RNA	Ribonucleic acid
SAE	Serious adverse event(s)
SAS	Statistical Analysis Software
SD	Standard deviation
SGOT	Serum glutamic-oxaloacetic transaminase
SGPT	Serum glutamic pyruvic transaminase
SOP	Standard Operating Procedure
SPM	Study Procedures Manual
SUSAR	Suspected, Unexpected, Serious Adverse drug Reaction
T	Infusion duration

t	Time of last observed quantifiable concentration
t½	Terminal phase half-life
τ	Dosing interval
tlag	Lag time before observation of drug concentrations in sampled matrix
tlast	Time of last quantifiable concentration
tmax	Time of occurrence of Cmax
ULN	Upper limit of normal
UK	United Kingdom
US	United States
Vd/F	Apparent volume of distribution after extravascular (e.g., oral) administration
WBC	White blood cells
WGS	Whole genome screen

Trademark Information

Trademarks of the GlaxoSmithKline group of companies
None

Trademarks not owned by the GlaxoSmithKline group of companies
Chiron RIBA
InForm
WinNonlin

1. I NTRODUCTION

1.1. Backgr ound

1.1.1. COPD and clinical management of the disease

COPD is a preventable and treatable disease which is characterised by airflow limitation that is not fully reversible. This airflow limitation is progressive and is associated with an abnormal inflammatory response of the lung to noxious particles or gases [Global Initiative for Chronic Obstructive Lung Disease guidelines [GOLD, 2008]). Pharmacological management of COPD attempts to reduce symptoms, improve quality of life, optimise lung function, reduce exacerbations and improve exercise tolerance.

Inhaled bronchodilators, including β_2-adrenoreceptor agonists and anticholinergics are the mainstays of therapy in patients diagnosed with COPD. Bronchodilator classes include drugs with a short duration of action (*e.g.* the β_2 agonist salbutamol, and the anticholinergic ipratropium bromide) or long duration of action (*e.g.* the β_2 agonist salmeterol, and the anticholinergic tiotropium bromide). The choice of therapeutic agent depends largely on individual response in terms of symptom relief and adverse effects, as all are effective at improving lung function as measured by FEV_1 (Forced Expiratory Volume).

Inhaled short acting, β_2 agonists have been the mainstays of therapy in patients diagnosed with COPD and have been proven to be effective and generally well tolerated. Stimulation of the β_2 adrenoreceptor in the lung relaxes bronchial smooth muscle cells which results in bronchodilation. Unwanted systemic side effects related to β_2 agonist treatment such as tachycardia, tremor, hyperglycaemia and hypokalaemia are generally mild and are limited by local administration and also tend to show tachyphylaxis.

Anticholinergic bronchodilators function by blocking endogenous cholinergic tone. Ipratropium bromide and oxitropium bromide are non-selective antagonists which act at the M_1, M_2 and M_3 receptors, and consequently may have bronchodilating activity through the M_1/M_3 receptors, and bronchoconstricting activity through the M_2 receptor. Tiotropium bromide is a more recent quaternary ammonium anticholinergic that shows kinetic selectivity for the M_1 and M_3 receptor subtypes over the M_2 subtype. All three of the above drugs are poorly absorbed, which limit the troublesome systemic effects observed with belladonna alkaloids.

1.1.2. GSK 573719

GSK573719 is a quinuclidine derivative which is a potent pan-active mAChR antagonist (anticholinergic) and is being developed for once daily treatment of COPD. It is a high affinity specific reversible mAChR antagonist that has rapid on and slow-off kinetics at the human M3 muscarinic receptor subtype. It has been shown to be an effective long acting bronchodilator in humans by inhalation.

Detailed information relating to non-clinical pharmacology, safety pharmacology, PK and metabolism, toxicology and other pre-clinical data can be found in the GSK573719 Investigator's Brochure (IB) [GlaxoSmithKline Document Number RM2006/00835/02].

To date, five clinical pharmacology studies have been conducted in a total of 92 healthy volunteers and 48 COPD subjects to investigate the safety, tolerability, pharmacokinetics and pharmacodynamics of single and repeat dose administration of GSK573719. Doses of up to 1000 μg once daily have been examined. A total of 75 healthy volunteers and 42 COPD subjects have been dosed with GSK573719. Completed studies comprise two single dose studies in healthy volunteer subjects (AC4105209) and COPD subjects (AC4108123), and two repeat dose studies in healthy volunteer subjects (AC4106889 and AC4110106). All doses have been well tolerated and no safety findings of concern have been seen in any of these studies. *In vitro* studies have shown that the predominant enzyme responsible for the metabolism of GSK573719 is cytochrome P450 isoenzyme 2D6 (CYP2D6). Therefore, one of the healthy volunteer studies, AC4110106, specifically evaluated subjects with normal and poor CYP2D6 activity.

Additionally, initial safety and pharmacokinetic data from an ongoing repeat dose study in subjects with COPD has been obtained. This study, AC4105211, was designed to evaluate the safety and tolerability of dosing with GSK573719 at 250 μg and 1000 μg once-daily for 7 days in subjects with COPD. However, due to a major protocol violation only the 250 μg dose was evaluated. The AC4105211 study has been amended to obtain the planned safety, tolerability, pharmacokinetic and pharmacodynamic data for the 1000 μg once-daily dose over 7 days in subjects with COPD.

1.1.3. GW6 42444

GW642444 is a novel, potent and selective long acting inhaled β_2 adrenoreceptor agonist (LABA), which is being developed for once-daily treatment of asthma and COPD. GW642444 is equipotent with salmeterol at β_2 adrenoceptors and is 1000 and 400 fold more selective for β_2 than β_1 and β_3 adrenoceptors, respectively. It has a longer duration of action and a faster onset of action than salmeterol in human bronchus and guinea pig trachea preparations and pre-clinical systemic exposure is limited by a low oral bioavailability (lower than salmeterol in the rat) and rapid breakdown. GW642444 has been engineered to be metabolically labile and is rapidly broken down in human microsomes into products with 100 fold less activity at the human β_2 adrenoceptor.

Clinical data have been obtained for GW642444 with a number of formulations; GW642444H, the alpha phenylcinnimate salt form with lactose alone; GW642444M, the triphenylacetate counter-ion with lactose and cellobiose octaacetate and GW642444M, with lactose and magnesium stearate (MgSt). To date, GW642444 has demonstrated a favourable safety profile in addition to sustained 24 hour bronchodilation (as assessed by trough FEV_1). Furthermore, GW642444 has demonstrated the potential for a broad therapeutic index. Please refer to the GW642444 Investigator's Brochure [GlaxoSmithKline Document Number SM2003/00028/06] for further details on the clinical studies conducted and completed to date.

1.1.4. GSK573719 and GW642444 concurrent dosing

This will be the first clinical study to evaluate the concurrent dosing of these two molecules.

An earlier study in healthy Japanese subjects (DB1112146) combining a similar muscarinic receptor antagonist GSK233705 and GW642444 [GlaxoSmithKline Document Number GH2008/00012/00] has recently been completed. This study was a randomised, double blind, placebo controlled, four-way crossover study and assessed for the first time, the safety, tolerability, PD and PK of GSK233705 and GW642444 when taken in combination from separate inhalers in healthy Japanese subjects.

Sixteen subjects were randomised in the study, to receive four possible treatments as single inhaled doses. Each subject received placebo, GSK233705 200 µg, GW642444 50 µg and GSK233705 200 µg /GW642444 50 µg combination once only. There were no deaths or serious adverse events (SAEs) reported during this study. A total of 33 AEs were reported by 12 subjects (75%). Out of these, two AEs were reported pre-treatment. All the adverse events (AEs) were mild to moderate in intensity. There were no withdrawals during the study and all the subjects completed four treatment periods.

There were no clinically significant or relevant changes in vital signs, haematology or biochemistry, which were attributed to study drug. Also there were no clinically significant12- lead electrocardiograms (ECG) or 24 hours (h) Holter ECG abnormalities observed during the study following dosing with study medication.

The results of the PK analysis showed that the ratio of the adjusted geometric means for AUC(0-6) and Cmax showed no clear evidence of a difference in GSK233705 systemic exposure when delivered as GSK233705 200 µg/GW642444 50 µg combination compared with GSK233705 alone. The ratio for Cmax showed some evidence of higher exposure when GSK233705 was administered in combination compared to when it was administered on its own.

In DB1111509, a similar study in healthy Caucasians, the treatment comparison ratios for both Cmax and AUC between combination versus individual treatment for both drugs (GSK233705 and GW642444) were close to unity for all 4 comparisons indicating no difference in systemic exposure [GlaxoSmithKline Document Number GM2007/00614/00.

1.1.5. M agnesium Stearate

Pre-clinical MgSt data are summarised in Appendix 1 of the IB for both GSK573719 and GW642444; GSK573719 Investigator Brochure [GlaxoSmithKline Document Number RM2006/00835/02] and GW642444 Investigator Brochure [GlaxoSmithKline Document Number SM2003/00028/06]. The proposed maximum daily dose of MgSt in GSK inhaled products is 0.25 mg/day based on inhalation of two 12.5 mg dry powder blisters each containing 1% w/w MgSt. The GSK573719 formulation has 0.6% w/w MgSt per inhalation; subjects will receive no more than one inhalation per day and so fall inside the proposed maximum daily dose. The GW642444 formulation has 1% w/w

MgSt per inhalation; subjects will also receive no more than one inhalation per day and so fall inside the proposed maximum daily dose. Subjects who are on the GSK573719 (500 µg) and GSK642444 (50 µg) arm will receive no more than one inhalation from each device and so would still fall inside the proposed maximum daily dose.

MgSt has FDA GRAS status as a food ingredient at concentrations of 0.01% to1%; an estimate of the average daily oral intake is of the order of 2.4 mg. MgSt is also widely used as a lubricant in tablet and capsule manufacture typically at levels of 0.2% to 0.5% w/w. MgSt has been approved for use as a dry powder excipient in inhaled respiratory medicines in 16 European countries as well as in Latin America, South Africa and New Zealand. One MgSt-containing respiratory product, Pulvinal (Beclomethasone Dipropionate), has been marketed in the UK for more than four years. In addition, Foradil, Certihaler, which also contains MgSt, received an approvable letter from the Food and Drug Association (FDA) in December 2004. GSK have analysed the contents of these two products and estimate that the total daily intake of MgSt in patients treated with them is in the range of 0.05 to 0.2 mg/day which is similar to the proposed maximum daily intake of MgSt from GSK products (0.25 mg/day).

1.2. Rational e

1.2.1. St udy Rationale

A dual LABA/LAMA combination therapy has not yet been developed for the treatment of COPD but is likely to be a valuable addition to currently available treatments. Both GSK573719 and GW642444 have the potential for once daily administration and the ability to combine them in the novel dry powder inhaler as a dual product means that these two molecules demonstrate promise as a combination treatment. This study will assess the safety and tolerability, pharmacodynamics and pharmacokinetics of this combination for the first time in healthy Japanese volunteers and will facilitate the participation of Japanese subjects in further clinical development of both the mono and dual therapy with a LAMA and LABA.

LABA and LAMA therapies are co-prescribed for COPD in clinical practice and the therapeutic benefit of concurrent therapy has been demonstrated. Studies in the literature show an additive effect on FEV_1 when a LABA and a LAMA are given in a combined treatment.

Both GSK573719 and GW642444 have been administered to healthy subjects and patients as a dry powder containing lactose and magnesium stearate.

1.2.2. D ose Rationale

GSK573719 is under development for once-daily dosing as a monotherapy product or as a component of fixed dose combination product with a LABA for the treatment of COPD. This section provides dose rationale for the fixed dose combination product of GSK573719 and GW642444M. As discussed in Section 1.1.4, no pharmacokinetic interaction between GW642444M and GSK233705, a similar M3 receptor antagonist,

was seen in study DB1112146. Therefore no interaction between GW642444M and GSK573719 is anticipated in the study described in this protocol and so the dose rationales for individual components are discussed separately.

1.2.2.1. G SK573719

The safety, tolerability, pharmacokinetics (PK) and pharmacodynamics (PD) of GSK573719 have been evaluated in five clinical studies in healthy subjects and COPD patients following single and repeat dose administration. The potential therapeutic once-daily dose of GSK573719 in COPD may be up to 500µg. This study will use 500µg as the dose, similar to both the repeat dose healthy volunteer study (AC4106889) and single dose COPD study (AC4106123). Analysis of GSK573719 in PK plasma samples from the single and repeat dose study in healthy volunteers (AC4106889) and from the single dose study in COPD patients (AC4108123), following administration of up to 1000µg demonstrated that Cmax occurred early at a median tmax of approximately five minutes and no subject exceeded pre-set systemic exposure based stopping criteria for Cmax and AUC. GSK573719 was well tolerated and no safety issues were identified in these studies.

1.2.2.2. G W642444M

The 50µg GW642444M dose selected for evaluation in this study is the highest dose evaluated in Phase IIb dose-ranging studies in patients with asthma and COPD. This dose has been shown to be well tolerated in previous studies with no clinically significant effect on heart rate. The GW642444M (GW642444 triphenylacetate salt formulation) with MgSt to be administered in this study was previously administered in a 14-day repeat dose study in healthy subjects (GW642444M doses 25, 50 and 100µg; study B2C108784), as a single dose to asthmatic patients (GW642444M doses 25, 50 and 100µg; study B2C106996) and as single doses to COPD patients (GW642444M doses 25, 50 and 100µg: study B2C110165). In B2C108784, GW642444M was well tolerated with an adverse event incidence similar to that seen with placebo. Higher doses of GW642444M produced typical beta-agonist mediated systemic effects including increased heart rate, blood glucose and QTc duration, none were considered clinically relevant, and there were no effects on blood pressure or potassium [GlaxoSmithKline Document Number GM2007/00052/00]. In the single dose study in male and female patients with persistent asthma (study B2C106996) GW642444M (25, 50 and 100µg) was well tolerated producing only small but statistically significant pharmacodynamic effects on physiological parameters typically at higher doses of long-acting beta-agonists. These were most frequently seen after administration of 100µg GW642444M.

A single dose of 50 µg GW642444M will be included in the present study to provide early information on the safety and tolerability when administered alone or in combination with GSK573719 and as described in Section 1.1.4. This dose has previously been safe and well tolerated in a healthy Japanese population both as a monotherapy and in combination with a novel LAMA GSK233705 in study DB112146 and shown that there is no increase in exposure or interaction as a result of combination with GSK233705, as discussed in Section 1.1.4.

1.3. Summary of Risk Management

- The study is being run at a clinical research unit based in a public hospital and has access to the hospital emergency cardiac arrest team and the hospital level 3 intensive care unit.
- Subjects will remain under medical supervision in the research facility until completion of the 24 hour post dose assessments in each treatment period, as a minimum. Subjects will not be discharged from the unit until the Investigator has reviewed all the available safety data.
- Subjects will be monitored during the post dose period for known effects of muscarinic antagonists and in particular tachycardia and arrhythmia. Acceptable limits for changes in these parameters have been established and safety criteria for consideration of subject withdrawal are included in the protocol, see Section 4.5.
- Subjects will be monitored during the post dose period for known effects of β_2 agonists and in particular tachycardia. Acceptable limits for changes in these parameters have been set and safety criteria for consideration of subject withdrawal are included in the protocol, see Section 4.5.

2. OBJECTIVE(S)

2.1. Primary

- To evaluate the safety and tolerability of GSK573719 (500µg) and GW642444 (50µg) administered as single inhaled doses and in combination (GSK573719 (500µg) and GW642444 (50µg)) in healthy Japanese subjects.

2.2. Secondary

- To evaluate the pharmacokinetics of GSK573219 (500µg) and GW642444 (50µg) administered as single inhaled doses and in combination (GSK573719 (500µg) and GW642444 (50µg)) in healthy Japanese subjects.

2.3. Exploratory

- To evaluate the effect of GSK573719 (500µg) and GW642444 (50µg) administered as single inhaled doses and in combination [GSK573719 (500µg) and GW642444 (50µg)] in healthy Japanese subjects on lung function parameters.

3. ENDPOINT(S)

3.1. Primary

General safety and tolerability endpoints: adverse events, heart rate, systolic and diastolic blood pressure, 12- lead ECG (QTc(B), QTc(F)), and lung function (FEV_1) and clinical laboratory safety tests. 24hr Holter monitoring will include: maximum and mean HR and arrhythmias.

The following endpoints will be derived for the resting heart rate, QTc(F) and QTc(B):
• Maximum value (0-4 hour)
• Weighted Mean (0-4 hour)

The following endpoints will be derived for blood potassium:
• Weighted mean (0-4 hour)
• Minimum values potassium (0-4 hour)

3.2. Secondary

- Plasma concentrations of GSK573719 and derived pharmacokinetic parameters.
- Plasma concentrations of GW642444 and derived pharmacokinetic parameters.

3.3. Exploratory

Serial FEV_1 measurements from 0h to 24 h post-dose.

4. INVESTIGATIONAL PLAN

4.1. Study Design Description

This design is a standard single centre, double-blind, placebo-controlled, four-way crossover, randomised, single dose study in healthy Japanese subjects.

All subjects will attend the unit for screening within 30 days of their first dosing period. The GSK573719 and GW642444 products will be delivered by using 2 monotherapy devices (one GSK573719 and GW642444 device). Therefore, each subject will receive a total of two devices; the second device will be a Placebo (Lactose Monohydrate) except when both GSK573719 and GW642444 are administered. Each subject will receive each of the following treatments once only

• Placebo and Placebo
• GSK573719 (500µg) and Placebo
• GW642444 (50µg) and Placebo
• GSK573719 (500µg) and GW642444 (50µg)

The order in which these treatments are administered will be in accordance with the randomisation schedule, and there will be a minimum washout period of 7 days between doses. For logistical reasons it is permissible for the wash-out period to be increased to a maximum of 28 days between dose periods.

This study includes a placebo arm to allow for a valid evaluation of adverse events attributable to treatment versus those independent of treatment. Each subject will be admitted to the unit in the day prior to Day 1 of each of the treatment periods and remain resident until all the 24 hour assessments have been completed. All subjects will attend the unit for a follow up visit 5-10 days following their final dose. The maximum duration (screening to follow-up) for randomised subjects will be about 10 weeks and will not exceed this unless washout period is extended for logistical reasons.

4.2. Discussion of Design

This study will assess the safety and tolerability, and pharmacokinetics of combining single doses of GSK573719 and GW642444 for the first time in healthy volunteers. In addition, it will be the FTIH study of a single dose of GSK573719 in healthy Japanese volunteers. DB1112146, a study in healthy Japanese [GlaxoSmithKline Document Number GH2008/00012/00], with a design similar to this study, using the drug combination GSK233705 (LAMA) and GW642444 (LABA) was recently completed. The results of the analysis showed that the ratio of the adjusted geometric means for AUC(0-6) and Cmax showed no clear evidence of a difference in GSK233705 systemic exposure when delivered as GSK233705 200 µg/GW642444 50 µg combination compared with GSK233705 alone. The ratio for Cmax showed some evidence of higher exposure when GSK233705 was administered in combination compared to when it was administered on its own. In DB1111509, a similar study in healthy Caucasians, the treatment comparison ratios for both Cmax and AUC between combination versus individual treatment for both drugs (GSK233705 and GW642444) were close to unity for all 4 comparisons indicating no difference in systemic exposure [GlaxoSmithKline Document Number GM2007/00614/00]. Cross-study comparison of the 2 studies indicated that systemic exposure in healthy Japanese volunteers was similar to that observed in healthy Caucasian volunteers for both drugs. For this reason, the single dose safety and pharmacokinetics study of the combination GSK573719 + GW642444 will be conducted in healthy Japanese volunteers only.

4.3. Treatment Assignment

Subjects will be assigned to one of the four treatment sequences in Table 1 which are based on a Williams Design in accordance with the randomization schedule generated by Discovery Biometrics, prior to the start of the study, using validated internal software.

A description of each regimen is provided in the Table 1:

Table 1 Treatment Sequences

Period 1	Period 2	Period 3	Period 4
Placebo and Placebo	GSK573719 (500 µg) and GW642444 (50 µg)	GSK573719 (500 µg) and Placebo	GW642444 (50 µg) and Placebo
GSK573719 (500 µg) and Placebo	Placebo and Placebo	GW642444(50 µg) and Placebo	GSK573719 (500 µg) and GW642444 (50 µg)
GW642444 (50 µg) and Placebo	GSK573719 (500 µg) and Placebo	GSK573719 (500 µg) and GW642444 (50 µg)	Placebo and Placebo
GSK573719 (500 µg) and GW642444 (50 µg)	GW642444(50 µg) and Placebo	Placebo and Placebo	GSK573719 (500 µg) and Placebo

A randomisation schedule will be produced with randomisation numbers 101-116. Each subject will be assigned a subject number and a randomisation number on the occasion of receiving their first dose of study medication. The randomisation number will equal subject number. Subjects who withdraw from the study may be replaced at the discretion of the Sponsor and Investigator. The replacement subject will be assigned to the same sequence (the replacement subject will receive the randomisation number of the withdrawn subject +100).

4.4. Investigational Product Dosage/Administration

For this study, GSK573719 inhalation powder is supplied in a novel dry powder inhaler containing a single strip, where each dose of GSK573719 delivers 500µg. GW642444 inhalation powder is supplied in a novel dry powder inhaler containing a single strip, where each dose of GW642444 delivers 50µg.

**Product name:**	GSK573719 GW	642444	GSK573719 and GW642444[3]	Placebo
**Formulation description:**	GSK573719 micronised drug blended with lactose monohydrate and magnesium stearate[1]	GW642444 micronised drug blended with lactose monohydrate and magnesium stearate[2]	GSK573719: micronised drug blended with lactose monohydrate and magnesium stearate[1] GW642444: micronised drug blended with lactose monohydrate and magnesium stearate[2]	Lactose monohydrate
**Dosage form:**	Novel Dry Powder Inhaler	Novel Dry Powder Inhaler	Novel Dry Powder Inhaler	Novel Dry Powder Inhaler
**Unit dose strength(s)**	GSK573719 500µg per blister	GW642444 50µg per blister	GSK573719 500µg per blister, GW642444 50µg per blister	Not applicable
**Route/ Administration/ Duration:**	Inhaled, once daily single doses	Inhaled, once daily single doses	Inhaled, once daily single doses	Inhaled, once daily single doses
**Manufacturer**	GSK GSK		GSK	GSK

1. Magnesium stearate 0.6% w/w of total drug product
2. Magnesium stearate 1 % w/w of total drug product
3. The combination product (GSK573719 and GW642444) will be administered to the subject by using 2 monotherapy devices (one GSK573719 and GW642444 device).

On the day of treatment, the allocated devices will be removed from refrigerated storage and allowed to equilibrate to ambient conditions for a minimum period of one hour prior to being administered to the subject.

4.5. Stopping Criteria

4.5.1. Stopping Safety Criteria

A subject will be withdrawn from the study if they meet any of the stopping criteria in this section.

Withdrawal of 3 subjects from the study, due to one of the criteria included in Section 4.5, would result in all remaining dose periods being cancelled and the study stopped for review.

4.5.1.1. Liver Chemistry Stopping Criteria

Liver chemistry threshold stopping criteria have been designed to assure subject safety and to evaluate liver event etiology during administration of investigational product and the follow-up period. Investigational product will be stopped if the following liver chemistry stopping criteria is met:

- ALT ≥ 3xULN

Refer to Section 13, Liver Chemistry Follow-up Procedures, for details of the required assessments if a subject meets the above criteria.

4.5.1.2. QTc Withdrawal Criteria

A subject that meets the criteria below will be withdrawn from the study.

- QTcB or QTcF > 500 msec or absolute change from baseline > 60 msec
- If subject has bundle branch block then criteria is QTcB or QTcF > 530 msec

These criteria are based on an average QTc value of triplicate ECGs. If an ECG demonstrates a prolonged QT interval, obtain 2 more ECGs over a brief period, and then use the averaged QTc values of the 3 ECGs to determine whether the subject should be discontinued from the study.

4.5.1.3. Further Safety Stopping Criteria

A subject will be withdrawn from the study if they meet the following stopping criteria:

- Subjects experience unacceptable adverse events related to study drug.
- Subjects demonstrate clinically significant and relevant ECG changes.
- Subjects demonstrate clinically significant and relevant changes in laboratory parameters
- Subjects experience a fall in FEV1 of ≥ 30% of pre-dose baseline within 2hr postdose.
- Subjects experience resting pulse increases of 40 bpm above the baseline resting heart rate or to a maximum of 130bpm at 2 successive measurements, a minimum of 5 minutes apart and if in the investigator's opinion, following a review of the subject's safety data and discussion with the GSK medical monitor, there are safety concerns.

For the purpose of stopping criteria the heart rate values obtained from the automated BP and pulse rate machine (Dinamap or equivalent) or from clinical examination will be utilised.

4.6. Time and Events Table

Table 2 Screening and Follow-Up Procedures

Procedure	Screening	Follow Up
Informed consent	X	
Medical History	X	
Physical examination (Full at screening, brief at Follow Up)	X	X
Clinical laboratory tests	X X	
Urinary DOA	X	
Alcohol & smoking urinary/breath tests	X	
Serology (hepatitis & HIV) screen	X	
BP and HR (***= In triplicate)	X*** X	
12-lead ECG (***= In triplicate)	X*** X	
Spirometry FEV1 and FVC (FVC only at screening) (***= In triplicate)	X***	X
Holter (24 hour)	X	
Inhaler training[1]	X	
SAE/AE monitoring	X	

1. May be performed pre-dose on Day 1 of first treatment period only if training inhaler devices are not available at screening visit

Table 3 Detailed Dose Period (1 to 4) Procedures

Procedure	Study Day (each dosing session)																	
	Day -1	Day 1																
		Pre-dose	0 H 0 M	0 H 5 M	0 H 15 M	0 H 30 M	0 H 45 M	1 h 0 M	1 H 30 M	2 H 0 M	3 H 0 M	4 H 0 M	5 H 0 M	6 H 0 M	8 H 0 M	12 H 0 M	16 H 0 m	24 H 0 M
In Clinic	X	X																
Clinical labs	X																	X
Blood PD sampling (potassium)		X		X	X	X		X		X	X	X						X
Alcohol & smoking urinary/breath tests	X																	
Urinary DOA	X																	
Dosing			X															
Vital signs (triplicate measurements)		X			X		X		X			X			X			X
Holter		X (0-24 H)																
12-Lead ECG (triplicate measurements)		X			X		X		X			X			X			X
Spirometry FEV1 (triplicate measurements)		X				X				X				X		X		X
Pharmacokinetic blood sampling (GSK573719 and GW642444)		X		X X X				X	X	X		X	X	X	X	X	X	X
SAE/AE/Concurrent medication questioning	X																	

5. STUDY POPULATION

5.1. Number of Subjects

A maximum of 16 subjects will be enrolled such that approximately 12 subjects complete dosing and critical assessments.

If subjects prematurely discontinue the study, additional subjects may be enrolled as replacement subjects and assigned to the same treatment sequence at the discretion of the Sponsor in consultation with the investigator.

5.2. Eligibility Criteria

5.2.1. Inclusion Criteria

A subject will be eligible for inclusion in this study only if all of the following criteria apply:

1. Healthy as determined by a responsible and experienced physician, based on a medical evaluation including medical history, physical examination, laboratory tests and cardiac monitoring. A subject with a clinical abnormality or laboratory parameters outside the reference range for the population being studied may be included only if the Investigator and the GSK Medical Monitor agree that the finding is unlikely to introduce additional risk factors and will not interfere with the study procedures.
2. Japanese ethnic origin defined as having been born in Japan with four ethnic Japanese grandparents and able to speak Japanese.
3. Male or female between 20 and 65 years of age inclusive, at the time of signing the informed consent.
4. A female subject is eligible to participate if she is of non-childbearing potential defined as pre-menopausal females with a documented tubal ligation or hysterectomy; or postmenopausal defined as 12 months of spontaneous amenorrhea [in questionable cases a blood sample with simultaneous follicle stimulating hormone (FSH) > 40 MlU/ml and estradiol < 40 pg/ml (<140 pmol/L) is confirmatory].
5. Male subjects must agree to use one of the contraception methods listed in Section 8.1. This criterion must be followed from the time of the first dose of study medication until completion of the follow-up visit.
6. Body weight ≥ 45 kg and BMI within the range 18-28 kg/m^2 inclusive.
7. AST, ALT, alkaline phosphatase and bilirubin ≤ 1.5xULN (isolated bilirubin >1.5xULN is acceptable if bilirubin is fractionated and direct bilirubin <35%).
8. Average QTcB or QTcF < 450 msec; or QTc < 480 msec in subjects with Bundle Branch Block taken from triplicate assessments at screening.

9. No clinically active and relevant abnormality on 12-lead ECG or 24h Holter ECG at screening.
10. Normal spirometry (FEV1 ≥ 80% of predicted, FEV1/FVC ≥ 70%).
11. Non-smokers (never smoked or not smoking for >6 months with <10 pack years history (Pack years = (cigarettes per day smoked/20) x number of years smoked))
12. A signed and dated written informed consent is obtained from the subject
13. The subject is capable of giving informed consent, which includes compliance with the requirements and restrictions listed in the consent form
14. Available to complete the study

5.2.2. Exclu sion Criteria

A subject will not be eligible for inclusion in this study if any of the following criteria apply:

1. Any clinically important abnormality identified at the screening medical assessment (physical examination/medical history), clinical laboratory tests, or ECG (12-lead). A 24hr Holter monitoring recording outside normal limits.
2. A history of breathing problems (i.e. history of asthmatic symptomatology, unless asthma in childhood that has now resolved and no longer requires maintenance therapy which should not be an exclusion).
3. A mean QTc(B) value at screening >450msec, or an ECG that is not suitable for QT measurements (e.g. LBBB or poorly defined termination of the T wave).
4. A history of elevated resting blood pressure or a mean blood pressure higher than 140/90 mmHg at screening.
5. A mean heart rate outside the range 40-90 bpm at screening.
6. The subject has a positive pre-study drug/alcohol screen. A minimum list of drugs that will be screened for include amphetamines, barbiturates, cocaine, opiates and benzodiazepines. The detection of drugs with a legitimate medical use would not necessarily be an exclusion to study participation. The detection of alcohol would not be an exclusion at screening but would need to be negative pre-dose and during the study.
7. A positive pre-study Hepatitis B surface antigen or positive Hepatitis C antibody result within 3 months of screening.
8. Current or chronic history of liver disease, or known hepatic or biliary abnormalities (with the exception of Gilbert's syndrome).
9. A positive test for HIV antibody.
10. History of regular alcohol consumption within 3months of the study defined as:
    - an average weekly intake of greater than 21 units or an average daily intake of greater than 3 units (males), or defined as an average weekly intake of greater

than 14 units or an average daily intake of greater than 2 units (females). Under Australian guidelines, one standard unit defined as 10g of ethanol, is equivalent to 250ml of full-strength beer, 470 ml of light-strength beer, 100 ml of wine and 30ml of spirits.

11. The subject has participated in a clinical trial and has received an investigational product within the following time period prior to the first dosing day in the current study: 30 days, 5 half-lives or twice the duration of the biological effect of the investigational product (whichever is longer).
12. Exposure to more than four new chemical entities within 12 months prior to the first dosing day.
13. Use of prescription or non-prescription drugs, including vitamins, herbal and dietary supplements (including St John's Wort) within 7 days (or 14 days if the drug is a potential enzyme inducer) or 5 half-lives (whichever is longer) prior to the first dose of study medication, unless in the opinion of the Investigator and GSK Medical Monitor the medication will not interfere with the study procedures or compromise subject safety.
14. History of sensitivity to any of the study medications, or components thereof or a history of drug or other allergy that, in the opinion of the investigator or GSK Medical Monitor, contraindicates their participation.
15. Where participation in the study would result in donation of blood or blood products in excess of 500 mL within a 56 day period.
16. Unwillingness or inability to follow the procedures outlined in the protocol.
17. Urinary cotinine levels indicative of smoking or history of regular use of tobacco- or nicotine-containing products prior to screening.
18. The subject is unable to use the novel dry powder inhaler correctly.
19. The subject has a known allergy or hypersensitivity to ipratropium bromide, tiotropium, atropine and any of its derivatives.
20. Any adverse reaction including immediate or delayed hypersensitivity to any β_2 agonist or sympathomimetic drug.
21. The subject has a known allergy or hypersensitivity to milk protein or the excipients lactose monohydrate and MgSt.
22. Subject is kept under regulatory of judicial order in an institution.
23. Any female of childbearing potential.
24. Subject is mentally or legally incapacitated.

5.2.3. Other Eligibility Criteria Considerations

To assess any potential impact on subject eligibility with regard to safety, the investigator must refer to the following document(s) for detailed information regarding warnings, precautions, contraindications, adverse events, and other significant data pertaining to the investigational product(s) being used in this study:

- GSK2573719 Investigator Brochure [GlaxoSmithKline Document Number RM2006/00835/02].
- GW642444 Investigator Brochure [GlaxoSmithKline Document Number SM2003/00028/06].

6. DATA ANALYSIS AND STATISTICAL CONSIDERATIONS

6.1. Hypotheses and Treatment Comparisons

Precision Estimation

This study is designed to estimate the effect of GSK573719 (500µg) and GW642444 (50µg) in combination relative to GSK573719 (500µg) alone; GW642444 (50µg) alone; and placebo and also each of the individual components relative to placebo on maximum heart rate (0-4h). No formal hypothesis will be tested. However, in order to provide precision estimates about the differences in the mean of the test treatment and the mean of the reference treatment for maximum heart rate, expected half widths for the confidence intervals have been produced (Table 4) .

The following treatment comparisons are of interest:

- GW642444 (50µg) and GSK573719 (500µg) in combination vs GSK573719 (500µg)
- GW642444 (50µg) and GSK573719 (500µg) in combination vs GW642444 (50µg)
- GW642444 (50µg) and GSK573719 (500µg) in combination vs Placebo
- GW642444 (50µg) alone vs Placebo
- GSK573719 (500µg) alone vs Placebo

The test treatment is either GW642444 (50µg) and GSK573719 (500µg) in combination or GW642444 (50µg) alone or GSK573719 (500µg) alone and the reference treatment is either GSK573719 (500µg) GW642444 (50µg) or Placebo depending on the comparison.

6.2. Sample Size Considerations

6.2.1. Sample Size Assumptions

Estimates of the within subject variation for maximum (0-4 hour) heart rate have been obtained from DB1112146, a placebo-controlled, four-way cross over study to assess the safety, tolerability, pharmacodynamics and pharmacokinetics of single inhaled dose of

GSK233705 and GW642444 as monotherapies and in combination in healthy Japanese subjects, AC4105209, a First Time in Human Study, which examined the safety, tolerability, pharmacodynamics and pharmacokinetics of single inhaled doses of GSK573719 in healthy male subjects, B2C10001, a First Time in Human Study to examine the safety, tolerability, pharmacodynamics and systemic pharmacokinetic profile of single inhaled doses of GW642444 in healthy male subjects, DB1111509, a placebo-controlled, four-way cross over study to assess the safety, tolerability, pharmacodynamics and pharmacokinetics of single inhaled doses of GSK233705 and GW642444 as monotherapies and in combination in healthy subjects, and HZA102940, a double-blind, placebo controlled, four-way crossover in healthy Japanese subjects which evaluated the safety, tolerability, pharmacodynamics and pharmacokinetics of GW642444 50mcg administered as separate inhaled single doses and in combination with GW685698, a novel inhaled corticosteroid (ICS). (Table 4)

With 12 subjects, assuming no difference between the treatment groups, and using the most conservative estimate of within subject standard deviation, it is estimated that the lower and upper bounds of the 95% confidence interval for the difference between test and reference treatments of maximum heart rate (0-4hours) will be within approximately 5.857 bpm of the point estimate.

Table 4 Precision Estimates for maximum heart rate (0-4hours)

Endpoint	Study	Within Subject standard deviation (bpm)	Expected ½ width of 95% Confidence Interval (bpm)
Maximum HR (0-4 hour)	DB1112146	3.164	2.638
AC	4105209	3.269	2.726
B2C	10001	4.783	3.988
D	B1111509	6.093	5.080
H	ZA102940	7.025	5.857

6.2.2. Sample Size Sensitivity

A sensitivity analysis was carried out to explore the effects of different variability estimates on the ½ width of the confidence interval for this study. These results are presented in Table 5.

Table 5 Precision Estimates for Various Variability Estimates

Within Subject Standard Deviation (bpm)	Expected ½ width of 95% Confidence Interval (bpm)
2 1.66	8
4 3.33	5
6 5.00	2
8 6.67	0
10 8.33	8
20 16.6	75

6.2.3. Sample Size Re-estimation

No sample size re-estimation will be performed.

6.3. Data Analysis Considerations

6.3.1. Int erim Analysis

No formal interim analysis is planned.

6.3.2. Final Analyses

6.3.2.1. Saf ety Analyses

Safety data will be presented in tabular and/or graphical format and summarized descriptively according to GSK's Integrated Data Standards Library (IDSL) standards.

For safety and tolerability data (including FEV_1), no formal hypotheses will be tested and no statistical analyses will be performed except for heart rate, QTc(F), QTc(B) and FEV_1.

Each of the maximum and weighted mean (0-4h) endpoints for heart rate, QTc(F) and QTc(B) will be statistically analysed using a mixed effects model. Subject level baseline (defined as the mean of the pre-dose measurements for each period), and period level baseline (calculated as the raw period baseline – subject level baseline) period and treatment group will be fitted as fixed effects and subject as a random effect; with comparisons occurring between GW642444 and GSK573719 combination and GW642444 alone, GSK573719 alone and placebo and also GW642444 versus placebo, and GSK573719 versus placebo.

Each of the minimum and weighted mean (0-4h) endpoints for blood potassium will be analysed using similar methodology to that described for the ECG and heart rate analyses where subject level baseline is defined as the pre-dose assessment on Day 1 for each period and period level baseline will be calculated as the raw period baseline – subject level baseline; with comparisons occurring between GW642444 and GSK573719

combination and GW642444 alone, GSK573719 alone and placebo and also GW642444 versus placebo, and GSK573719 vs placebo.

6.3.2.2. Expl oratory Pharmacodynamic Analyses

An exploratory analysis on FEV_1 data may be conducted across serial timepoints (up to 24 hr) using repeated measures modelling.

6.3.2.3. Pharmacoki netic Analyses

Pharmacokinetic analysis will be the responsibility of the Clinical Pharmacokinetics Modeling and Simulation Department, CPDM, GlaxoSmithKline. Plasma concentration-time data for GSK573719 and GW642444 will be analyzed by non-compartmental methods with WinNonlin [version 4.1]. Calculations will be based on the actual sampling times recorded during the study. From the plasma concentration-time data, the following pharmacokinetic parameters will be determined, as data permit: maximum observed plasma concentration (Cmax), time to Cmax (tmax), area under the plasma concentration-time curve [AUC(0-t) and AUC(0-∞)], and apparent terminal phase half-life (t1/2).

Pharmacokinetic data will be presented in graphical and/or tabular form and will be summarized descriptively. All pharmacokinetic data will be stored in the Archives, GlaxoSmithKline Pharmaceuticals, R&D.

Statistical analyses of the pharmacokinetic parameter data will be the responsibility of Discovery Biometrics, GlaxoSmithKline.

Systemic exposure of GW642444 and GSK573719 will be evaluated when administered in combination compared to administration alone using $\log_e$- transformed AUC(0-8) and Cmax in a mixed effects model. Period and treatment will be fitted as fixed effects and subject will be fitted as a random effect. If AUC(0-∞) is non calculable due to NQ data in terminal phase, AUC(0-t) will be used with t being the latest time at which concentration is >NQ for all treatments (for example AUC(0-4) or AUC(0-6) etc). The following treatment comparisons will be conducted:

- GW642444 (50µg) and GSK573719 (500µg) – GW642444 (50µg)
- GW642444 (50µg)and GSK573719 (500µg) – GSK573719 (500µg)

6.3.2.4. Pharmacokinetic/Pharmacodynamic Analyses of Safety Parameters

If changes in heart rate and/or potassium are observed then the relationship between these parameters and GW642444 or GSK573719 systemic exposure will be explored graphically in the first instance and where a relationship is observed this will be evaluated further with PK/PD model.

7. STUDY ASSESSMENTS AND PROCEDURES

This section lists the parameters of each planned study assessment. The exact timing of each assessment is listed in the Time and Events Tables (Section 4.6). Detailed procedures for obtaining each assessment are provided in the Study Procedures Manual (SPM).

Whenever vital signs, 12-lead ECGs and blood draws are scheduled for the same nominal time, the 12-lead ECG will be obtained first followed by vital signs and then any blood draws.

Prior to being enrolled in the study and any study procedures being performed, subjects must sign the informed consent.

Each subject will undergo screening procedures within 30 days of the administration of their first dose of study medication and will have a follow up visit between 5 to 10 days inclusive post their last dose. See Table 2 for assessments performed at the screening and follow-up visits.

A generic ethics approved screening protocol may be used to identify potential subjects. Data from this generic screen may be used for the purpose of this study provided that it is within 60 days of the first dosing administration. Any assessments that are not included in this generic screen and are specific for this study must be performed once ethics and regulatory approvals have been received.

The timing and number of planned study assessments including safety, pharmacokinetic, or pharmacodynamic assessments may be altered during the course of the study based on newly available data (e.g. to obtain data closer to the time of peak plasma concentrations) to ensure appropriate monitoring. The change in timing or addition of time points for any planned study assessments must be approved and documented by GSK, but this will not constitute a protocol amendment. The IRB/IEC will be informed of any safety issues that require alteration of the safety monitoring scheme.

No more than 500 mL of blood will be collected over the duration of the study, including any extra assessments that may be required.

7.1. Demogr aphic/Medical History Assessments

The following demographic parameters will be captured: date of birth, gender, race and ethnicity.

Medical/medication/alcohol and smoking history will be assessed as related to the eligibility criteria listed in Section 5.2.

7.2. Safet y

Planned time-points for all safety assessments are listed in the Time and Events Table (Section 4.6). Additional time points for safety tests such as vital signs, ECG and laboratory safety tests may be added during the course of the study based on newly available data to ensure appropriate safety monitoring.

Physical Exams

- A complete physical examination will include assessments of the head, eyes, ears, nose, throat, skin, thyroid, neurological, lungs, cardiovascular, abdomen (liver and spleen), lymph nodes and extremities. Height and weight will also be measured and recorded.
- A brief physical examination will include assessments of the skin, lungs, cardiovascular system, and abdomen (liver and spleen).

Vital Signs

Vital sign measurements will include systolic and diastolic blood pressure and pulse rate.

- Resting heart rate and blood pressure measurements will be made at screening, at various occasions throughout the treatment periods, and, at the follow-up visit as outlined in Section 4.6 Time and Events Table.
- Three measurements will be taken at screening and pre-dose, 1 – 5mins apart. The mean value recorded pre-dose will be classed as baseline.
- Subject will be required to rest in the semi-recumbent position at ~45 degrees for at least 5mins before each reading.
- Heart rate and blood pressure will be measured with an automatic measuring device.

Electrocardiogram (ECG)

Twelve-lead ECGs will be obtained at each time-point during the study using an ECG machine that automatically calculates the heart rate and measures PR, QRS, QT, and QTc intervals and derives QTc(B). Refer to Section 4.5.1.2 for QTc withdrawal criteria and additional QTc readings that may be necessary.

Twelve-lead ECGs will be performed at screening, at various occasions throughout the treatment periods and at the follow-up visit as outlined in Section 4.6 Time and Events Table.

Three measurements will be taken at screening and pre-dose, 1-5mins apart. The mean value recorded pre-dose will be classed as baseline.

ECG measurements will be made (1-5mins apart for repeat measurements) with the subject in a semi-recumbent at 45 degrees having rested in this position for at least 5 mins before each reading.

Extreme changes in room temperature should be avoided as far as possible (the subjects should be comfortable at all times in appropriate clothing).

Hot and cold drinks and food should be avoided where possible 30mins before an ECG measurement.

Care should be taken to ensure that the electrodes are placed in the positions dictated by standard ECG methodology.

Paper ECG traces will be recorded at a standard paper speed of 25mm/sec and gain of 1mV/10mm. VR, PR, QRS, QT, $QTc_{(B)}$ will be calculated automatically. ECGs will be stored electronically for manual measurement of intervals, if necessary.

ECG-related stopping criteria for subjects are described in Section 4.5.1.2.

Holter Monitoring

24hr Holter monitoring will be conducted at times indicated in Time and Events Tables (Section 4.6). Holter tapes will be analysed locally by the site and results obtained and reviewed within 48hr (two working days).

During the screening procedure and study a standard Holter monitor will be used (in order to exclude subjects with underlying cardiac arrhythmogenicity).

N.B. During the treatment periods, Holter monitors should only be switched on immediately prior to dosing (up to 15mins pre-dose) so as to capture Holter ECG data from the 24hr period following dosing.

The following summary data will be transcribed into the CRF:

- Maximum and mean (0-24hr) heart rate
- Arrhythmias

Analysis of the Holter tapes will be performed by the site.

Clinical Laboratory Assessments

Hematology, clinical chemistry, urinalysis and additional parameters to be tested are listed below.

Laboratory samples are not required to be taken fasted, however, if an out of normal clinical range glucose results is obtained this should be repeated with a fasted sample.

Hematology

Platelet Count	*RBC Indices: Automated*	*WBC Differential:*
RBC Count	MCV	Neutrophils
WBC Count (absolute)	MCH	Lymphocytes
Hemoglobin MCHC		Monocytes
Hematocrit		Eosinophils
		Basophils

Clinical Chemistry

BUN	Potassium	AST (SGOT)	Total and direct bilirubin
Creatinine	Calcium	ALT (SGPT)	Uric Acid
Glucose		GGT	Albumin
Sodium		Alkaline phosphatase	Total Protein

Routine Urinalysis

Specific gravity
pH, glucose, protein, blood and ketones by dipstick
Microscopic examination (if blood or protein is abnormal)

Urinalysis will be performed over times indicated in Time and Events Tables (Section 4.6). Full disclosures will be maintained as part of the subject's source documents and will be reviewed in detail by qualified site staff. Only dates and times and clinical result need be recorded in the CRF, unless the assessment is abnormal and clinically significant, then it will be recorded as an AE.

Other tests (Screening Visit only)

HIV (if local SOPs require)
Hepatitis B (HBsAg)
Hepatitis C (Hep C antibody -- if second generation Hepatitis C antibody positive, a hepatitis C antibody Chiron RIBA immunoblot assay should be reflexively performed **on the same sample** to confirm the result)
FSH and estradiol (as needed in post-menopausal women only)
Alcohol and drug screen (to include at minimum: amphetamines, barbiturates, cocaine, opiates, cannabinoids and benzodiazepines).

Further details regarding these tests can be obtained from the SPM.

Blood Potassium

In addition to the potassium performed as part of the standard laboratory safety assessments, this will also be monitored to assess the pharmacodynamic effects of GW642444 at the time points listed in Section 4.6, Time and Events Tables.

Blood serum samples for potassium will be analysed by the safety laboratory.

Further details will be provided in the SPM.

Lung Function Tests

Forced expiratory volume in 1 second (FEV_1) and forced vital capacity (FVC) will be measured at screening and FEV_1 during each treatment period and at follow-up as outlined in Time and Events Tables (Section 4.6).

Lung function tests will be recorded whilst the subject is in a sitting position (if taken whilst the subject is on the bed, their legs should be over the edge). At screening, the FEV_1 and FVC will be measured.

All lung function tests will be repeated, until three technically acceptable measurements have been made. All three measurements for FEV_1 and FVC will be recorded in the subjects' CRF. FVC will only be measures at screening and FEV_1/FVC ratio will be determined using the highest FEV_1 and the highest FVC values.

7.3. Alcohol, Cotinine and Drugs of Abuse Tests

Alcohol (urine or breath) test, tobacco (urine cotinine or breath) and urine drugs of abuse tests will be performed at screening and on admission into the unit for any dosing period and may be carried out occasionally, on other occasions, throughout the study.

The drugs of abuse to be screened for are listed in Section 5.2 and in the SPM.

7.4. Pregnan cy

7.4.1. Action to be taken if pregnancy occurs in a female partner of a male study subject

The investigator will attempt to collect pregnancy information on any female partner of a male study subject who becomes pregnant while participating in this study. The investigator will record pregnancy information on the appropriate form and submit it to GSK within 2 weeks of learning of the partner's pregnancy. The partner will also be followed to determine the outcome of the pregnancy. Information on the status of the mother and child will be forwarded to GSK. Generally, follow-up will be no longer than 6 to 8 weeks following the estimated delivery date. Any premature termination of the pregnancy will be reported.

7.5. Pharma cokinetics

7.5.1. Blood Sample Collection

Blood samples for pharmacokinetic analysis of GSK573719 and GW642444 will be collected at the time points indicated in Section 4.6, Time and Events Table. The actual date and time of each blood sample collection will be recorded. The timing of PK samples may be altered and/or PK samples may be obtained at additional time points to ensure thorough PK monitoring.

Details of PK blood sample collection (including volume to be collected), processing, storage and shipping procedures are provided in the Study Procedures Manual (SPM).

7.5.2. Sample Analysis

Plasma analysis will be performed under the management of Worldwide Bioanalysis, DMPK, GlaxoSmithKline. Concentrations of GSK573719 and GW642444 will be determined in plasma samples using the currently approved analytical methodology. Raw data will be stored in the GLP Archives, GlaxoSmithKline.

8. LIFESTYLE AND/OR DIETARY RESTRICTIONS

8.1. Contrac eption Requirements

To prevent pregnancy in a female partner or to prevent exposure of any partner to the investigational product from a male subject's semen, male subjects must use one of the following contraceptive methods:

- Abstinence, defined as sexual inactivity consistent with the preferred and usual lifestyle of the subject. Periodic abstinence (e.g. calendar, ovulation, symptothermal, post-ovulation methods) and withdrawal are not acceptable methods of contraception.
- Condom (during non-vaginal intercourse with any partner - male or female) **OR**
- Condom and occlusive cap (diaphragm or cervical/vault caps) plus spermicidal agent (foam/gel/film/cream/suppository) *(during sexual intercourse with a female)*

8.2. Meals and Dietary Restrictions

Subjects will be required to follow the restrictions below:

- Fasting 4hr pre-dose to 4hr post dose
- Water will be allowed freely, except for 1hour either side of dosing on all dosing occasions.
- Subjects will receive standardised meals during their in-house periods; no other food is allowed.

- Subjects must refrain from grapefruit or grapefruit juice containing products from 7 days pre-dose (Day 1) of the first treatment period until discharge from the unit (Day 2) of the final treatment period.
- Subjects will receive standardised Japanese meals during their in-house periods as described in the SPM.

8.3. Caffeine, Alcohol, and Tobacco

- During each dosing session, subjects will abstain from ingesting caffeine- or xanthine-containing products (e.g. coffee, tea, cola drinks, chocolate) for 24 hours prior to the start of dosing until collection of the final pharmacokinetic and or pharmacodynamic sample during each session.
- During each dosing session, subjects will abstain from alcohol for 24 hours prior to the start of dosing until collection of the final pharmacokinetic and or pharmacodynamic sample during each session.
- Use of tobacco products is not allowed from the start of dosing and until after the final follow-up visit.
- Subjects must refrain from all recreational drugs throughout the study (screening to follow-up). Drugs of abuse tests may be performed randomly throughout the study to check this. A positive result will lead to exclusion from the study.

8.4. A ctivity

Subjects will abstain from strenuous exercise for 48 hours prior to each blood collection for clinical laboratory tests. Subjects may participate in light recreational activities during studies (e.g., watch television, read*)*.

9. CONCOMITANT MEDICATIONS AND NON-DRUG THERAPIES

9.1. Permitt ed Medications

Except for simple analgesics, over the counter preparations including vitamins, herbal remedies, and dietary supplements will not be permitted for 7 days before each study day, unless it is judged by the investigator not to compromise subject safety or influence the outcome of the study.

The Investigator (or designated study physician) must be informed as soon as possible about any medication taken from the time of screening until the end of the clinical phase of the study (follow-up visit).

All concomitant medications taken during the study will be recorded in the CRF. The minimum requirement is that drug name and the dates of administration are to be recorded.

9.2. Prohibited Medications

Other than simple analgesics, subjects must abstain from taking prescription or non-prescription drugs (including vitamins and dietary or herbal supplements), within 7 days (or 14 days if the drug is a potential enzyme inducer) or 5 half-lives (whichever is longer) prior to the first dose of study medication until completion of the follow-up visit, unless in the opinion of the Investigator and sponsor the medication will not interfere with the study.

9.3. Non-Drug Therapies

Subjects must abstain from taking any vitamins, herbal and dietary supplements within 7 days (or 14 days if the drug is a potential enzyme inducer) or 5 half-lives (whichever is longer) prior to the first dose of study medication until completion of the follow-up visit, unless in the opinion of the Investigator and sponsor the medication will not interfere with the study.

10. COMPLETION OR EARLY WITHDRAWAL OF SUBJECTS

10.1. Subject Completion

A completed subject is one who has completed all phases of the study including the follow-up visit.

The end of the study is defined as the last subject's last visit.

10.2. Subject Withdrawal Criteria

Refer to Section 4.5 for dose adjustment/stopping criteria based on safety/PK/PD criteria.

A subject may withdraw from investigational product at any time at his/her own request, or may be withdrawn at any time at the discretion of the investigator for safety, behavioral or administrative reasons.

10.3. Subject Withdrawal Procedures

10.3.1. Subject Withdrawal from Study

For subjects withdrawn form the investigational product, follow-up procedures should be performed. Relevant pages of the CRF should be completed by the investigator.

10.4. Treatment After the End of the Study

Subjects will not receive any additional treatment after completion of the study because only healthy volunteers are eligible for study participation.

10.5. Screen and Baseline Failures

Data for screen and baseline failures will be collected in source documentation at the site but will not be transmitted to GSK. The reason(s) for excluding these subjects will be documented. The source documentation (including ICF) for all such failures will be made available to the monitor for monitoring.

11. INVESTIGATIONAL PRODUCT(S)

Investigational product dosage and administration details are listed in Section 4.4.

11.1. Blinding

This will be a double-blind study.

The investigator or treating physician may unblind a subject's treatment assignment only in the case of an emergency, when knowledge of the study treatment is essential for the appropriate clinical management or welfare of the subject. Whenever possible, the investigator must first discuss options with the GSK Medical Monitor or appropriate GSK study personnel before unblinding the subject's treatment assignment. If this is impractical, the investigator must notify GSK as soon as possible, but without revealing the treatment assignment of the unblinded subject, unless that information is important for the safety of subjects currently in the study. The date and reason for the unblinding must be recorded in the appropriate data collection tool.

GSK's Global Clinical Safety and Pharmacovigilance (GCSP) staff may unblind the treatment assignment for any subject with an SAE. If the SAE requires that an expedited regulatory report be sent to one or more regulatory agencies, a copy of the report, identifying the subject's treatment assignment, may be sent to clinical investigators in accordance with local regulations and/or GSK policy.

11.2. Packaging and Labeling

The contents of the label will be in accordance with all applicable regulatory requirements.

The site staff will be responsible for carrying out dispensing activities of the subject treatment packs; this will be performed by the pharmacist according to the randomisation code provided by GSK and double checked by another pharmacist.

11.3. Preparation/Handling/Storage/Accountability

No special preparation of investigational product is required.

Investigational product must be dispensed or administered according to procedures described herein. Only subjects enrolled in the study may receive investigational product. Only authorized site staff may supply or administer investigational product. All

investigational products must be stored in a secure area with access limited to the investigator and authorized site staff. Investigational product is to be stored under physical conditions consistent with investigational product-specific requirements. Maintenance of a temperature log (manual or automated) is required.

In order to maintain the blind in this study, all treatment packs will be stored refrigerated at 2-8^0 C. On the day of treatment, the allocated devices will be removed from refrigerated storage and allowed to equilibrate to ambient conditions for a minimum period of one hour prior to being administered to the subject. The investigator, institution, or the head of the medical institution (where applicable) is responsible for investigational product accountability, reconciliation, and record maintenance. The investigator or the head of the medical institution (where applicable), or designated site staff (e.g., storage manager, where applicable) must maintain investigational product accountability records throughout the course of the study. The responsible person(s) will document the amount of investigational product received from and returned to GSK and the amount supplied and/or administered to and/or returned by subjects. The required accountability unit for this study will be inhalers. Discrepancies are to be reconciled or resolved. Procedures for final disposition of unused investigational product are listed in the SPM.

Investigational product is not expected to pose significant occupational safety risk to site staff under normal conditions of use and administration. A Material Safety Data Sheet (MSDS)/equivalent document describing occupational hazards and recommended handling precautions either will be provided to the investigator, where this is required by local laws, or is available upon request from GSK.

However, precautions are to be taken to avoid direct skin contact, eye contact, and generating aerosols or mists. In the case of unintentional occupational exposure notify the monitor, medical monitor and/or study manager.

Precaution will be taken to avoid direct contact with the investigational product. A Material Safety Data Sheet (MSDS) describing occupational hazards and recommended handling precautions will be provided to the investigator.

11.4. A ssessment of Compliance

When subjects are dosed at the study site, they will receive investigational products directly from the investigator or designee, under medical supervision. The date and time of each dose administered in the clinic will be recorded in the source documents. The dose of investigational product(s) and study participant identification will be confirmed at the time of dosing by a member of the study site staff other than the person administering the investigational product. Study site personnel will examine the counter on the subject's novel dry powder inhaler to ensure that the investigational product was dispensed.

11.5. Treatment of Investigational Product Overdose

An overdose for this study will be considered as any dose of study drug more than the planned dose on each dosing occasion. In the event of an overdose, there are no recommended medications or non-drug therapies for treatment. Management should be supportive and the investigator should use his/her clinical judgement in treating any overdose situation. Subjects experiencing such adverse events will be followed up clinically until the event has resolved.

GSK does not recommend specific treatment for an overdose. The investigator will use clinical judgment to treat any overdose.

12. ADVERSE EVENTS (AE) AND SERIOUS ADVERSE EVENTS (SAE)

The investigator or site staff is responsible for detecting, documenting and reporting events that meet the definition of an AE or SAE.

AEs will be collected from the start of Investigational Product and until the follow-up contact. Medical occurrences that begin prior to the start of investigational product but after obtaining informed consent may be recorded on the Medical History/Current Medical Conditions CRF.

SAEs will be collected over the same time period as stated above for AEs. However, any SAEs assessed as related to study participation (e.g. investigational product, protocol-mandated procedures, invasive tests, or change in existing therapy) or related to a GSK concomitant medication will be recorded from the time a subject consents to participate in the study up to and including any follow-up contact. All SAEs will be recorded and reported to GSK within 24 hours, as indicated in Section 12.7.

Investigators are not obligated to actively seek AEs or SAEs in former study participants. However, if the investigator learns of any SAE, including a death, at any time after a subject has been discharged from the study, and he/she considers the event reasonably related to the investigational product or study participation, the investigator would promptly notify GSK.

12.1. Definition of Adverse Events

An AE is any untoward medical occurrence in a patient or clinical investigation subject, temporally associated with the use of a medicinal product, whether or not considered related to the medicinal product.

Note: An AE can therefore be any unfavorable and unintended sign (including an abnormal laboratory finding), symptom, or disease (new or exacerbated) temporally associated with the use of a medicinal product.

Events meeting the definition of an AE **include**:

- Any abnormal laboratory test results (hematology, clinical chemistry, or urinalysis) or other safety assessments (e.g., ECGs, radiological scans, vital signs measurements), including those that worsen from baseline, and felt to be clinically significant in the medical and scientific judgement of the investigator.
- Exacerbation of a chronic or intermittent pre-existing condition including either an increase in frequency and/or intensity of the condition.
- New conditions detected or diagnosed after investigational product administration even though it may have been present prior to the start of the study.
- Signs, symptoms, or the clinical sequelae of a suspected interaction.

- Signs, symptoms, or the clinical sequelae of a suspected overdose of either investigational product or a concomitant medication (overdose per se will not be reported as an AE/SAE).

Events that **do not** meet the definition of an AE include:

- Any clinically significant abnormal laboratory findings or other abnormal safety assessments that are associated with the underlying disease, unless judged by the investigator to be more severe than expected for the subject's condition.
- The disease/disorder being studied, or expected progression, signs, or symptoms of the disease/disorder being studied, unless more severe than expected for the subject's condition.
- Medical or surgical procedure (e.g., endoscopy, appendectomy); the condition that leads to the procedure is an AE.
- Situations where an untoward medical occurrence did not occur (social and/or convenience admission to a hospital).
- Anticipated day-to-day fluctuations of pre-existing disease(s) or condition(s) present or detected at the start of the study that do not worsen.

12.2. Definition of Serious Adverse Events

If an event is not an AE per Section 12.1, then it can not be an SAE even if serious conditions are met (e.g., hospitalization for signs/symptoms of the disease under study, death due to progression of disease, etc).

An SAE is any untoward medical occurrence that, at any dose:

a. Results in death

b. Is life-threatening

   NOTE: The term 'life-threatening' in the definition of 'serious' refers to an event in which the subject was at risk of death at the time of the event. It does not refer to an event, which hypothetically might have caused death, if it were more severe.

c. Requires hospitalization or prolongation of existing hospitalization

   NOTE: In general, hospitalization signifies that the subject has been detained (usually involving at least an overnight stay) at the hospital or emergency ward for observation and/or treatment that would not have been appropriate in the physician's office or out-patient setting. Complications that occur during hospitalization are AEs. If a complication prolongs hospitalization or fulfills any other serious criteria, the event is serious. When in doubt as to whether "hospitalization" occurred or was necessary, the AE should be considered serious.

   Hospitalization for elective treatment of a pre-existing condition that did not worsen from baseline is not considered an AE.

d. Results in disability/incapacity, or

NOTE: The term disability means a substantial disruption of a person's ability to conduct normal life functions. This definition is not intended to include experiences of relatively minor medical significance such as uncomplicated headache, nausea, vomiting, diarrhea, influenza, and accidental trauma (e.g. sprained ankle) which may interfere or prevent everyday life functions but do not constitute a substantial disruption.

e. Is a congenital anomaly/birth defect

f. Medical or scientific judgment should be exercised in deciding whether reporting is appropriate in other situations, such as important medical events that may not be immediately life-threatening or result in death or hospitalization but may jeopardize the subject or may require medical or surgical intervention to prevent one of the other outcomes listed in the above definition. These should also be considered serious. Examples of such events are invasive or malignant cancers, intensive treatment in an emergency room or at home for allergic bronchospasm, blood dyscrasias or convulsions that do not result in hospitalization, or development of drug dependency or drug abuse.

g. Is associated with liver injury **and** impaired liver function defined as:

- ALT ≥ 3xULN, and
- total bilirubin ≥ 2xULN or INR > 1.5.

NOTES:

Bilirubin fractionation should be performed if testing is available. If fractionation is unavailable, urinary bilirubin is to be measured via dipstick (a measurement of direct bilirubin, which would suggest liver injury).

If INR is obtained, the value is to be recorded on the SAE form. INR elevations >1.5 suggest severe liver injury.

12.3. Method of Detecting AEs and SAEs

Care will be taken not to introduce bias when detecting AEs and/or SAEs. Open-ended and non-leading verbal questioning of the subject is the preferred method to inquire about AE occurrence. Appropriate questions include:

- "How are you feeling?"
- "Have you had any (other) medical problems since your last visit/contact?"
- "Have you taken any new medicines, other than those provided in this study, since your last visit/contact?"

12.4. Recording of AEs and SAEs

When an AE/SAE occurs, it is the responsibility of the investigator to review all documentation (e.g., hospital progress notes, laboratory, and diagnostics reports) relative to the event. The investigator will then record all relevant information regarding an AE/SAE in the appropriate data collection tool.

It is not acceptable for the investigator to send photocopies of the subject's medical records to GSK in lieu of completion of the GSK, AE/SAE data collection tool. However, there may be instances when copies of medical records for certain cases are requested by GSK. In this instance, all subject identifiers, with the exception of the subject number, will be blinded on the copies of the medical records prior to submission of to GSK.

The investigator will attempt to establish a diagnosis of the event based on signs, symptoms, and/or other clinical information. In such cases, the diagnosis will be documented as the AE/SAE and not the individual signs/symptoms.

12.5. Evaluating AEs and SAEs

12.5.1. Assessment of Intensity

The investigator will make an assessment of intensity for each AE and SAE reported during the study and will assign it to one of the following categories:

Mild: An event that is easily tolerated by the subject, causing minimal discomfort and not interfering with everyday activities.

Moderate: An event that is sufficiently discomforting to interfere with normal everyday activities.

Severe: An event that prevents normal everyday activities.

An AE that is assessed as severe will not be confused with an SAE. Severity is a category utilized for rating the intensity of an event; and both AEs and SAEs can be assessed as severe. An event is defined as 'serious' when it meets at least one of the pre-defined outcomes as described in the definition of an SAE.

12.5.2. Assessment of Causality

The investigator is obligated to assess the relationship between investigational product and the occurrence of each AE/SAE. A "reasonable possibility" is meant to convey that there are facts/evidence or arguments to suggest a causal relationship, rather than a relationship cannot be ruled out. The investigator will use clinical judgment to determine the relationship. Alternative causes, such as natural history of the underlying diseases, concomitant therapy, other risk factors, and the temporal relationship of the event to the investigational product will be considered and investigated. The investigator will also

consult the Investigator Brochure (IB) and/or Product Information, for marketed products, in the determination of his/her assessment.

There may be situations when an SAE has occurred and the investigator has minimal information to include in the initial report to GSK. However, **it is very important that the investigator always make an assessment of causality for every event prior to the initial transmission of the SAE data to GSK.** The investigator may change his/her opinion of causality in light of follow-up information, amending the SAE data collection tool accordingly. The causality assessment is one of the criteria used when determining regulatory reporting requirements.

12.6. Follow-up of AEs and SAEs

After the initial AE/SAE report, the investigator is required to proactively follow each subject at subsequent visits/contacts. All AEs and SAEs will be followed until resolution, until the condition stabilizes, until the event is otherwise explained, or until the subject is lost to follow-up.

The investigator is obligated to perform or arrange for the conduct of supplemental measurements and/or evaluations as may be indicated or as requested by GSK to elucidate as fully as possible the nature and/or causality of the AE or SAE. The investigator is obligated to assist. This may include additional laboratory tests or investigations, histopathological examinations or consultation with other health care professionals. If a subject dies during participation in the study or during a recognized follow-up period, the investigator will provide GSK with a copy of any post-mortem findings, including histopathology.

New or updated information will be recorded in the originally completed data collection tool. The investigator will submit any updated SAE data to GSK within the designated reporting time frames.

12.7. Prompt Reporting of SAEs to GSK

Once the investigator determines that an event meets the protocol definition of an SAE, the SAE will be reported to GSK **within 24 hours**. Any follow-up information on a previously reported SAE will also be reported to GSK within 24 hours.

If the investigator does not have all information regarding an SAE, he/she will not wait to receive additional information before notifying GSK of the event and completing the appropriate data collection tool. The investigator will always provide an assessment of causality at the time of the initial report as described in Section 12.5.2, Assessment of Causality.

The primary mechanism for reporting SAEs to GSK will be the electronic data collection tool (e.g., InForm system). If the electronic system is unavailable for greater than 24 hours, the site will use the paper SAE data collection tool and fax it to the GSK Medical Monitor (Dr Glenn Crater) and to the GSK Australia named safety contact (Debra Ong);

contact details for these personnel are provided in the SPM. Then the site will enter the serious adverse event data into the electronic system as soon as it becomes available.

After the study is completed at a given site, the electronic data collection tool (e.g., InForm system) will be taken off-line to prevent the entry of new data or changes to existing data. If a site receives a report of a new SAE from a study participant or receives updated data on a previously reported SAE after the electronic data collection tool has been taken off-line, the site can report this information on a paper SAE form or to their GSK protocol contact by telephone.

GSK contacts for SAE receipt can be found at the beginning of this protocol on the Sponsor/Medical Monitor Contact Information page.

12.8. Regulatory Reporting Requirements For SAEs

Prompt notification of SAEs by the investigator to GSK is essential so that legal obligations and ethical responsibilities towards the safety of subjects are met.

GSK has a legal responsibility to notify both the local regulatory authority and other regulatory agencies about the safety of a product under clinical investigation. GSK will comply with country specific regulatory requirements relating to safety reporting to regulatory authorities, IRBs/IECs and investigators.

Investigator safety reports are prepared for suspected unexpected serious adverse reactions according to local regulatory requirements and GSK policy and are forwarded to investigators as necessary. An investigator who receives an investigator safety report describing an SAE(s) or other specific safety information (e.g., summary or listing of SAEs) from GSK will file it with the IB and will notify the IRB/IEC, if appropriate according to local requirements.

13. LIVER CHEMISTRY FOLLOW-UP PROCEDURES

Refer to the diagram in Appendix 1 for a visual presentation of the procedures listed below.

The procedures listed below are to be followed if a subject meets the liver chemistry stopping criteria defined in Section 4.5.1.1:

- Immediately and permanently withdraw the subject from investigational product
- Notify the GSK medical monitor within 24 hours of learning of the abnormality to confirm the subject's investigational product cessation and follow-up.
- Complete the "Safety Follow-Up Procedures" listed below.
- Complete the liver event case report forms. If the event also meets the criteria of an SAE (see Section 12.2), the SAE data collection tool will be completed separately with the relevant details.

- Upon completion of the safety follow-up permanently withdraw the subject from the study and do not rechallenge with investigational product.

Safety Follow-Up Procedures for subjects with ALT ≥3xULN:

- Monitor subjects weekly until liver chemistries (ALT, AST, alkaline phosphatase, bilirubin) resolve, stabilize or return to within baseline values.

Safety Follow-Up Procedures for subjects with ALT ≥3xULN and bilirubin ≥2xULN:

- This event is considered an SAE (see Section 12.2). Serum bilirubin fractionation should be performed if testing is available. If fractionation is unavailable, urinary bilirubin is to be measured via dipstick (a measurement of direct bilirubin, which would suggest liver injury).
- Make every reasonable attempt to have subjects return to the clinic within 24 hours for repeat liver chemistries, additional testing, and close monitoring (with specialist or hepatology consultation recommended).
- Monitor subjects twice weekly until liver chemistries (ALT, AST, alkaline phosphatase, bilirubin) resolve, stabilize or return to within baseline values.

In addition, for all subjects with ALT ≥3xULN, every attempt must be made to also obtain the following:

- Viral hepatitis serology including:
  - Hepatitis A IgM antibody.
  - Hepatitis B surface antigen and Hepatitis B Core Antibody (IgM).
  - Hepatitis C RNA.
  - Cytomegalovirus IgM antibody.
  - Epstein-Barr viral capsid antigen IgM antibody (or if unavailable, obtain heterophile antibody or monospot testing).
  - Hepatitis E IgM antibody (if subject resides outside the USA or Canada, or has traveled outside USA or Canada in past 3 months).
- Blood sample for pharmacokinetic (PK) analysis, obtained within 24 hours or 3 half-lives of last dose. Record the date/time of the PK blood sample draw and the date/time of the last dose of investigational product prior to blood sample draw on the CRF. If the date or time of the last dose is unclear, provide the subject's best approximation. If the date/time of the last dose can not be approximated OR a PK sample can not be collected in the time period indicated above, **do not obtain a PK sample**. Instructions for sample handling and shipping are included in the SPM.
- Serum creatine phosphokinase (CPK) and lactate dehydrogenase (LDH).
- Fractionate bilirubin, if total bilirubin ≥ 2xULN.
- Assess eosinophilia

- Record the appearance or worsening of clinical symptoms of hepatitis (fatigue, nausea, vomiting, right upper quadrant pain or tenderness, fever, rash or eosinophilia) on the AE CRF.
- Record use of concomitant medications, acetaminophen, herbal remedies, other over the counter medications, or putative hepatotoxins on the Concomitant Medications CRF.
- Record alcohol use on the Liver Events CRF.

The following are required for subjects with ALT ≥3xULN **and** bilirubin ≥2xULN but are optional for other abnormal liver chemistries:

- Anti-nuclear antibody, anti-smooth muscle antibody, and Type 1 anti-liver kidney microsomal antibodies.
- Liver imaging (ultrasound, magnetic resonance, or computerized tomography) to evaluate liver disease.
- The Liver Imaging and/or Liver Biopsy CRFs are also to be completed if these tests are performed.

14. STUDY CONDUCT CONSIDERATIONS

14.1. Regulatory and Ethical Considerations, Including the Informed Consent Process

GSK will obtain favorable opinion/approval to conduct the study from the appropriate regulatory agency in accordance with any applicable country-specific regulatory requirements prior to a site initiating the study in that country.

The study will be conducted in accordance with all applicable regulatory requirements.

The study will also be conducted in accordance with "good clinical practice" (GCP), all applicable subject privacy requirements, and, the guiding principles of the 2008 Declaration of Helsinki. This includes, but is not limited to, the following:

- IRB/IEC review and favorable opinion/approval to conduct the study and of any subsequent relevant amended documents
- Written informed consent (and any amendments) to be obtained for each subject before participation in the study
- Investigator reporting requirements (e.g. reporting of AEs/SAEs/protocol deviations to IRB/IEC)

14.2. Quality Control (Study Monitoring)

In accordance with applicable regulations including GCP, and GSK procedures, GSK monitors will contact the site prior to the start of the study to review with the site staff the protocol, study requirements, and their responsibilities to satisfy regulatory, ethical, and

GSK requirements. When reviewing data collection procedures, the discussion will also include identification, agreement and documentation of data items for which the "CRF" will serve as the source document.

GSK will monitor the study and site activity to verify that the:

- Data are authentic, accurate, and complete.
- Safety and rights of subjects are being protected.
- Study is conducted in accordance with the currently approved protocol and any other study agreements, GCP, and all applicable regulatory requirements.

The investigator and the head of the medical institution (where applicable) agrees to allow the monitor direct access to all relevant documents

14.3. Qualit y Assurance

To ensure compliance with GCP and all applicable regulatory requirements, GSK may conduct a quality assurance audit. Regulatory agencies may also conduct a regulatory inspection of this study. Such audits/inspections can occur at any time during or after completion of the study. If an audit or inspection occurs, the investigator and institution agree to allow the auditor/inspector direct access to all relevant documents and to allocate his/her time and the time of his/her staff to the auditor/inspector to discuss findings and any relevant issues.

14.4. Study and Site Closure

Upon completion or premature discontinuation of the study, the monitor will conduct site closure activities with the investigator or site staff, as appropriate, in accordance with applicable regulations including GCP, and GSK procedures.

In addition, GSK reserves the right to temporarily suspend or prematurely discontinue this study at any time for reasons including, but not limited to, safety or ethical issues or severe non-compliance. If GSK determines such action is needed, GSK will discuss this with the investigator or the head of the medical institution (where applicable), including the reasons for taking such action. When feasible, GSK will provide advance notification to the investigator or the head of the medical institution, where applicable, of the impending action prior to it taking effect.

If the study is suspended or prematurely discontinued for safety reasons, GSK will promptly inform investigators or the head of the medical institution (where applicable) and the regulatory authorities of the suspension or premature discontinuation of the study and the reason(s) for the action. If required by applicable regulations, the investigator or the head of the medical institution (where applicable) must inform the IRB/IEC promptly and provide the reason for the suspension or premature discontinuation.

14.5. Record s Retention

Following closure of the study, the investigator or the head of the medical institution (where applicable) must maintain all site study records, except for those required by local regulations to be maintained by someone else, in a safe and secure location. The records must be maintained to allow easy and timely retrieval, when needed (e.g., audit or inspection), and, whenever feasible, to allow any subsequent review of data in conjunction with assessment of the facility, supporting systems, and staff. Where permitted by local laws/regulations or institutional policy, some or all of these records can be maintained in a format other than hard copy (e.g., microfiche, scanned, electronic); however, caution needs to be exercised before such action is taken. The investigator must assure that all reproductions are legible and are a true and accurate copy of the original, and meet accessibility and retrieval standards, including re-generating a hard copy, if required. Furthermore, the investigator must ensure there is an acceptable back-up of these reproductions and that an acceptable quality control process exists for making these reproductions.

GSK will inform the investigator of the time period for retaining these records to comply with all applicable regulatory requirements. The minimum retention time will meet the strictest standard applicable to that site for the study, as dictated by any institutional requirements or local laws or regulations, or GSK standards/procedures; otherwise, the retention period will default to 15 years.

The investigator must notify GSK of any changes in the archival arrangements, including, but not limited to, archival at an off-site facility or transfer of ownership of the records in the event the investigator leaves the site.

14.6. Provision of Study Results and Information to Investigators

Where required by applicable regulatory requirements, an investigator signatory will be identified for the approval of the clinical study report. The investigator will be provided reasonable access to statistical tables, figures, and relevant reports and will have the opportunity to review the complete study results at a GSK site or other mutually-agreeable location.

Upon completion of the clinical study report, GSK will ensure public disclosure of the clinical trial research results via the GSK Clinical Trial Register according to the GSK SOP. The investigator is encouraged to share the summary results with the study subjects, as appropriate.

GSK will provide the investigator with the randomization codes for their site after the statistical analysis for the entire study has been completed.

14.7. Data Management

GSK Data Management will identify and implement the most effective data acquisition and management strategy for each clinical trial protocol and deliver datasets which support the protocol objectives. Subject data will be entered into GSK defined CRFs and combined with data provided from other sources (e.g. diary data, laboratory data) in a validated data system. Subject initials will not be transmitted to GSK for inclusion in the datasets. Clinical data management will be performed in accordance with applicable GSK standards and data cleaning procedures with the objective of removing errors and inconsistencies in the data which would otherwise impact on the analysis and reporting objectives, or the credibility of the Clinical Study Report. Adverse events and concomitant medications terms will be coded using validated dictionaries. Original CRFs will be retained by GSK, while the investigator will retain a copy.

15. REFERENCES

GlaxoSmithKline Document Number GH2008/00012/00 A single centre, randomised, placebo-controlled, four-way cross over study to assess the safety, tolerability, pharmacodynamics and pharmacokinetics of single inhaled doses of GSK233705 and GW642444 as monotherapies and in combination in healthy Japanese subjects. Effective date 26 Aug 2008

GlaxoSmithKline Document Number GM2007/00052/00. A randomised, single-dose, dose-ascending, double-blind, placebo-controlled, 5-way crossover study to investigate the safety, tolerability, pharmacokinetics and pharmacodynamics of inhaled doses of GW642444M with magnesium stearate in asthmatic patients. Date 20- Dec-2007

GlaxoSmithKline Document Number GM2007/00614/00 A single centre, randomised, placebo-controlled, four-way cross over study to assess the safety, tolerability, pharmacodynamics and pharmacokinetics of single inhaled doses of GSK233705 and GW642444 as monotherapies and in combination in healthy subjects. Effective date 27 Mar 2008

GlaxoSmithKline Document Number RM2006/00835/02, Investigator's Brochure GSK573719. Report Date 1-Apr-2009.

GlaxoSmithKline Document NumberSM2003/00028/06, Investigator's Brochure for GW642444. Report Date 29-Jan-2008.

Global Initiative for Chronic Obstructive Lung Disease [GOLD]: Global Strategy for the Diagnosis, Management, and Prevention of Chronic Obstructive Pulmonary Disease; updated 2008.

APPENDICES

APPENDIX 1: LIVER SAFETY ALGORITHMS

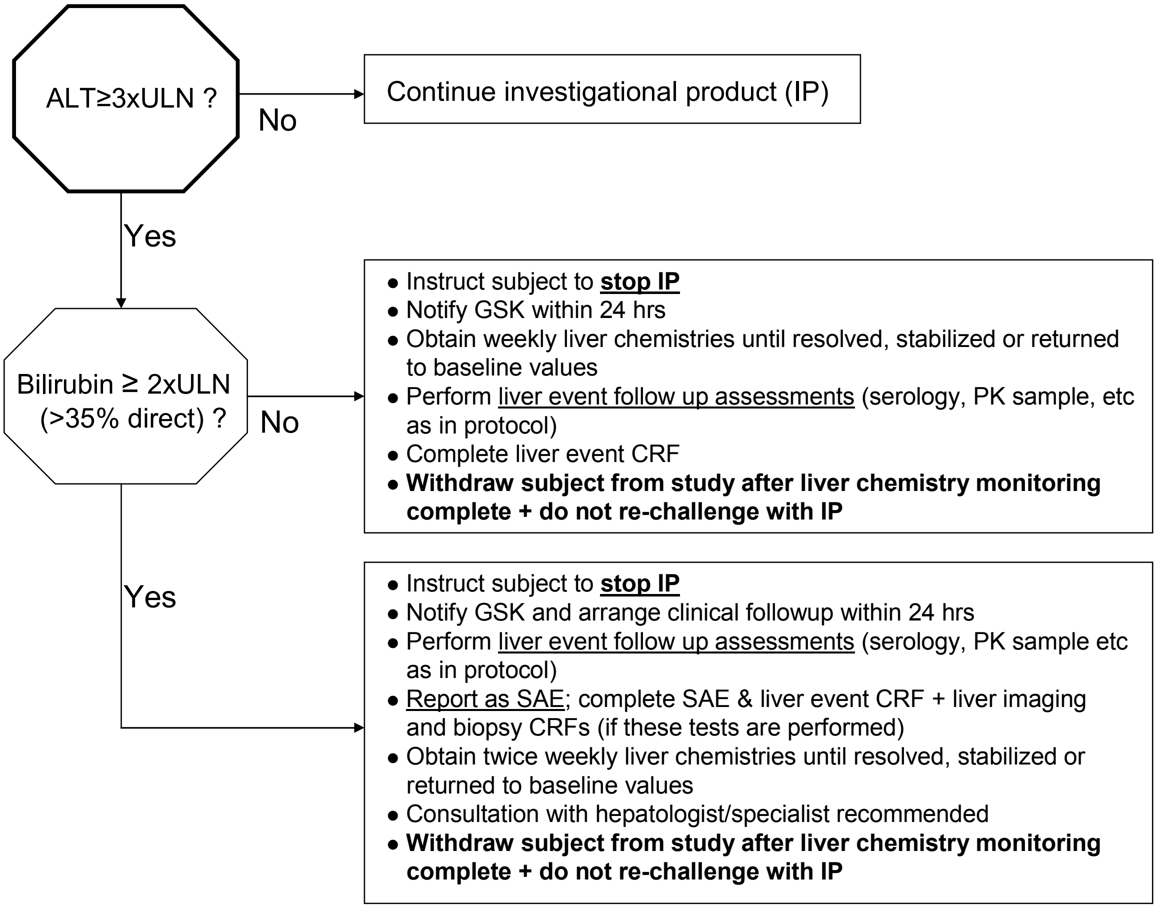

Supplement: Protocol S1 — Trial Protocol. (PDF) [file pone.0050716.s002.pdf]
